# A paracrine-to-autocrine shunt of GREM1 fuels colorectal cancer metastasis via ACVR1C

**DOI:** 10.1186/s12943-025-02554-w

**Published:** 2026-01-24

**Authors:** Huaixiang Zhou, Qunlong Jin, Zhang Fu, Yanming Yang, Yunfei Gao, Niu Wang, Bo Zhao, Long Gui, Jiang Li, Zijing Zhu, Ying Zhang, Yulong He, Ying Zhang, Shouqing Luo, Li Fu, Xudong Wu, Guihua Wang, Zhiming Xu, Huiliang Li, Junjing Zhang, Xuetong Shen, Tao Wang, Youheng Jiang, Ningning Li

**Affiliations:** 1 Tomas Lindahl Nobel Laureate Laboratory, The Seventh Affiliated Hospital of Sun Yat-sen University, Shenzhen, China; 2 Inner Mongolia Honder College of Arts and Sciences, Hohhot, China; 3 Digestive Diseases Center, Guangdong Provincial Key Laboratory of Digestive Cancer Research, The Seventh Affiliated Hospital of Sun Yat-sen University, Shenzhen, China; 4 Department of Geriatrics, The Seventh Affiliated Hospital of Sun Yat-sen University, Shenzhen, China; 5 Department of Otolaryngology, The Seventh Affiliated Hospital of Sun Yat-sen University, Shenzhen, China; 6 School of Medicine, Shenzhen Campus of Sun Yat-sen University, Shenzhen, China; 7 Institute of Bio-Architecture and Bio-Interactions, Shenzhen Medical Academy of Research and Translation, Shenzhen, China; 8 Clinical Big Data Research Center, The Seventh Affiliated Hospital of Sun Yat-sen University, Shenzhen, China; 9 Key Laboratory of Epigenetic Regulation and Intervention, Institute of Biophysics, Chinese Academy of Sciences, Beijing, China; 10 The Francis Crick Institute, London, UK; 11 Peninsula Medical School, Faculty of Health, University of Plymouth, Plymouth, UK; 12 Guangdong Key Laboratory for Genome Stability & Disease Prevention, Department of Pharmacology and Shenzhen International Cancer Center, Shenzhen University Medical School, Shenzhen University, Shenzhen, China; 13 The Province and Ministry Co-sponsored Collaborative Innovation Center for Medical Epigenetics, Key Laboratory of Immune Microenvironment and Disease (Ministry of Education), Department of Cell Biology, School of Basic Medical Sciences, Tianjin Medical University, Tianjin, China; 14 GI Cancer Research Institute, Tongji Hospital, and State Key Laboratory for Diagnosis and Treatment of Severe Zoonotic Infectious Diseases, Huazhong University of Science and Technology, Wuhan, China; 15 Future Medical Center, Shenzhen University of Advanced Technology, Shenzhen, China; 16 Wolfson Institute for Biomedical Research, Division of Medicine, Faculty of Medical Sciences, University College London, London, UK; 17 Department of Hepato-Biliary Surgery, Inner Mongolia Key Laboratory of Allergic Diseases, Hohhot First Hospital, Hohhot, China; 18 Institute of Cancer Research, Shenzhen Bay Laboratory, Shenzhen, China; 19 Shenzhen Clinical Research Center for Gastroenterology (Gastrointestinal Surgery), Shenzhen, China

**Keywords:** Colorectal cancer, GREM1–ACVR1C axis, Paracrine-to-autocrine shift, Signaling autonomy, EMT

## Abstract

**Background:**

Tumor cells typically rely on paracrine stromal signals to guide malignant behavior, yet whether they gain signaling autonomy and thereby reduce microenvironment dependency during metastasis remains unclear.

**Methods:**

Gremlin 1 (GREM1) and activin A receptor type 1C (ACVR1C) expression levels and cellular distribution were analyzed by immunohistochemistry, immunofluorescence (IF) staining, and single-cell transcriptomics in colorectal cancer (CRC) specimens across stages I–IV. The GREM1–ACVR1C interaction was identified and validated by interaction proteomics, co-immunoprecipitation, IF, and microscale thermophoresis (MST). Functional roles of the GREM1–ACVR1C axis in epithelial–mesenchymal transition (EMT) and metastasis were examined by transcriptomic profiling, pathway analysis, immunoblotting, reverse transcription quantitative PCR (RT–qPCR), scratch and transwell assays, and genetically engineered and xenograft mouse models. An inhibitory peptide targeting the GREM1–ACVR1C interface was designed and evaluated.

**Results:**

While GREM1 remains restricted to stromal cells in earlier-stage (I–III) CRC, its ectopic expression in tumor epithelium increases markedly in stage IV. Mechanistically, we identify ACVR1C as a direct, high-affinity epithelial receptor for GREM1. Their interaction, independent of canonical transforming growth factor β receptor (TGFβR) and bone morphogenetic protein (BMP) signaling, activates SMAD2/3, which in turn induces the transcription of *SNAI1* and *GREM1*, thereby establishing a self-sustaining autocrine loop that amplifies EMT. Disrupting this loop via stromal GREM1 deletion, epithelial *ACVR1C* knockdown, kinase inhibition, or a novel GREM1-blocking peptide targeting the GREM1–ACVR1C binding interface significantly impairs CRC metastasis in vivo. Remarkably, while stromal GREM1 is required to initiate this loop, epithelial-derived GREM1 is sufficient to maintain metastatic progression. Clinically, epithelial GREM1 or ACVR1C expression predicts aggressive disease and poor survival.

**Conclusions:**

Our findings define a paradigm wherein CRC cells hijack the stromal factor GREM1 to establish a tumor-autonomous GREM1–ACVR1C autocrine loop. This loop licenses signaling independence, drives sustained EMT, and represents a novel, actionable vulnerability in advanced CRC.

**Supplementary Information:**

The online version contains supplementary material available at 10.1186/s12943-025-02554-w.

## Introduction

Distant metastasis remains the leading cause of death among patients with colorectal cancer (CRC) [1]. A complex and dynamic interplay between tumor epithelial cells and various non-malignant components of the tumor microenvironment (TME) is recognized as a central driver of tumor initiation, progression, and phenotypic plasticity [2]. However, once tumor cells detach from the primary site to metastasize, they inevitably lose the continuous support of the local TME—including stromal cells and localized signaling cues. This abrupt interruption of paracrine support represents a major bottleneck that restricts most tumor cells from successfully establishing distant colonies [3]. This phenomenon raises a fundamental question about how a minority of tumor cells sustain metastasis-associated phenotypes without ongoing microenvironmental support. The capacity to sustain malignant behavior without external cues can be viewed as a form of “signaling autonomy”, consistent with the classic cancer hallmark of “self-sufficiency in growth signals” described by Hanahan and Weinberg [4]. While signaling autonomy has been extensively explored in the context of proliferation and survival, it remains unclear whether metastasizing tumor cells can acquire autonomous control over the programs that sustain their migratory capacity.

Within the TME, cancer-associated fibroblasts (CAFs) are among the most active stromal components, driving tumor initiation and progression through bidirectional interactions with cancer cells [5]. Among the highly heterogeneous CAF population, a Gremlin 1 (GREM1)-expressing subset has gained attention for promoting tumor progression via paracrine GREM1 secretion in CRC [6–8] and breast cancer [9]. Intriguingly, GREM1 expression is not restricted to stromal CAFs. In pancreatic [10] and prostate cancers [11], tumor epithelial cells can express and secrete GREM1 to regulate their own phenotypic plasticity, and epithelial-specific GREM1 is markedly upregulated in Hereditary Mixed Polyposis Syndrome (HMPS) [12]. Nonetheless, whether such epithelial GREM1 expression occurs in sporadic CRC and contributes to cancer cell plasticity or metastasis remains unknown. Studies, including ours, show that modulating GREM1 expression in CRC cell lines affects cell migration and epithelial–mesenchymal transition (EMT)-related phenotypes [13, 14]. Yet, whether CRC epithelial cells can “hijack” stromal GREM1 signals to ultimately activate their own GREM1 expression for malignant progression remains an open question. At the molecular level, GREM1 is known as a canonical antagonist of bone morphogenetic protein (BMP) signaling [15]. Recent studies indicate that GREM1 also possesses cytokine-like functions, binding noncanonical receptors, including VEGFR2 [16], FGFR1 [11], and EGFR [17], suggesting that it may influence cancer cell behavior through multiple signaling routes. However, it remains unclear whether GREM1 drives signaling autonomy in CRC via these receptors or others yet unidentified.

In this study, we identify a paracrine-to-autocrine shunt of GREM1 in CRC, driven by the newly recognized receptor activin A receptor type 1C (ACVR1C) [18], a transforming growth factor β (TGFβ) superfamily type I receptor, thereby leading to SMAD2/3 phosphorylation. This self-sustaining loop confers tumor cells with signaling autonomy and metastatic potential, uncovering a previously unrecognized mechanism of tumor evolution and a potential approach for therapeutic intervention in CRC.

## Results

### Ectopic expression of GREM1 during CRC progression

In the gut, GREM1 marks a subpopulation of fibroblasts in both normal [19] and tumor tissues [6]. Using *Grem1-CreER*^*T2*^;*Rosa-mTmG* mice [19], we confirmed that Grem1^+^ cells are exclusively confined to the stromal compartment, and distributed along the intestinal isthmus and adjacent to α-SMA^+^ myofibroblasts [20], two months post-tamoxifen (TMX) injection (Figures S1A–C). Extending this observation to humans, we found that GREM1 was likewise absent from epithelial cells in normal intestinal tissues, but sporadically expressed in stromal cells (Figure S1D), suggesting a conserved stromal specificity. Similarly, in human stage I–III CRC samples, GREM1 staining co-localized with VIMENTIN (VIM, a stromal cell marker, encoded by *VIM*) [20, 21] and fibroblast activation protein (FAP, an activated fibroblast marker) [20], but was mutually exclusive with β-CATENIN (β-CAT, a CRC cell marker, encoded by *CTNNB1*) [22], CD68 (a macrophage marker) [21], or α-SMA (a myofibroblast marker) (Figures S1E–J). These findings confirm that GREM1 is a bona fide stromal factor and GREM1^+^ stromal cells are a subtype of CAFs, potentially contributing to CRC progression [6, 8].

**Fig. 1:**
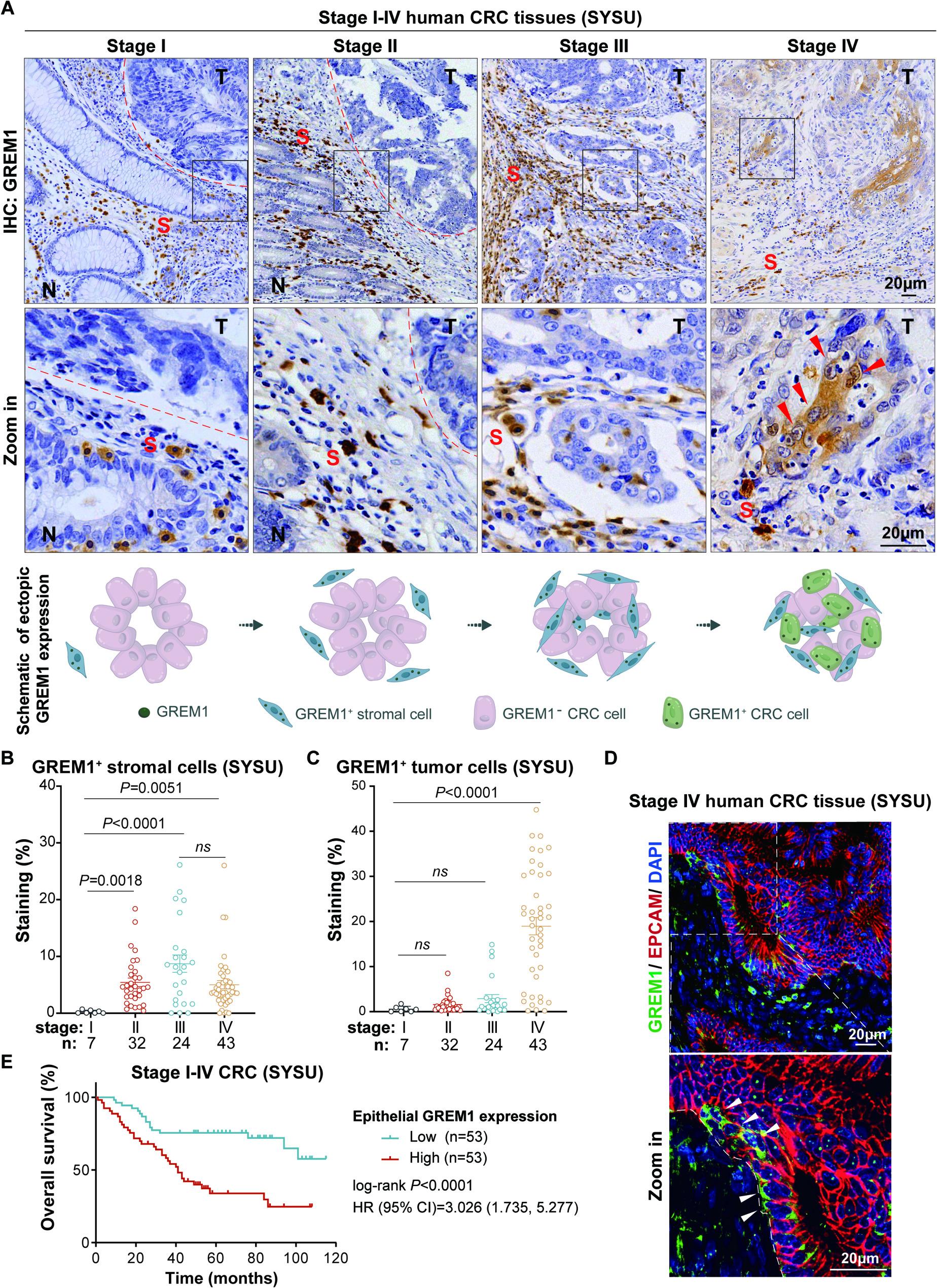
Ectopic expression of GREM1 protein in CRC. **A**, Representative images of GREM1 immunohistochemical (IHC) staining (Upper) on human primary CRC samples and illustration of GREM1 expression pattern (Lower). The red dashed line separates tumor from adjacent normal area. N: normal areas; T: tumoral areas; S: stromal areas; Red arrows: GREM1^+^ CRC cells. **B**, **C**, Quantification of GREM1^+^ stromal cell infiltration (**B**) and GREM1 expression in cancer cells (**C**) across stage I–IV primary CRC tissues from Sun Yat-sen University (SYSU). **D**, Representative immunofluorescence (IF) images of GREM1 and EPCAM staining in stage IV human primary CRC tumors. The white dashed line separates epithelial and mesenchymal cells. White arrows: GREM1^+^ CRC cells. **E**, Kaplan–Meier survival curves of 106 CRC patients from SYSU stratified by GREM1 levels in cancer cells. Patients were divided into high and low groups based on the cohort median. For **B** and **C**, data are mean ± s.e.m. *P* values were calculated using one-way ANOVA with Bonferroni’s multiple-comparison test (**B**, **C**). Significance was determined using a two-sided log-rank test. HR, hazard ratio (**E**). *ns*: not significant

To systematically investigate the distribution and clinical significance of GREM1, we first performed immunohistochemical (IHC) staining on 106 human primary CRC samples spanning all four stages. We observed a stage-dependent redistribution of GREM1^+^ cells: in early-stage tumors (stage I–II), GREM1^+^ stromal cells were predominantly restricted to the peritumoral stroma; in stage III, these cells more frequently infiltrated the tumor parenchyma; in stage IV, infiltration of GREM1^+^ stromal cells showed a decreasing trend (with no statistically significant difference compared to stage III). Notably, in stage IV tumors, strong GREM1 staining emerged in a subset of tumor epithelial regions (Figs. 1A–C). These findings suggest a potential shift of GREM1 expression from stroma to epithelium during tumor progression.

To complement this, we analyzed publicly available single-cell RNA sequencing (scRNA-seq) datasets covering human CRC samples from stages I–IV. Consistent with IHC data, *GREM1* expression was primarily detected in fibroblasts and epithelial cells (Figures S2A–C). Stage-specific analysis revealed a marked upregulation of *GREM1* in fibroblasts at stages III and IV (Figure S2D). Strikingly, *GREM1*^*+*^ epithelial cells were detected almost exclusively in stage IV tumors (Figure S2E), supporting the notion that ectopic GREM1 expression by tumor cells is a late event in CRC progression. This observation was further validated by colocalization of GREM1^+^ cells with an epithelial marker, epithelial cell adhesion molecule (EPCAM) [21], in stage IV tumor cells (Fig. 1D). Finally, survival analysis demonstrated that patients with high GREM1 expression in tumor cells had significantly shorter overall survival compared to those with low expression (Fig. 1E). Taken together, these results reveal that while GREM1^+^ CAFs are present throughout CRC progression, GREM1^+^ tumor epithelial cells emerge predominantly in advanced CRC, indicating that GREM1 expression, initially restricted to stromal cells, is progressively co-opted by tumor epithelial cells as CRC advances.

### ACVR1C is a novel GREM1 receptor in CRC

**Fig. 2:**
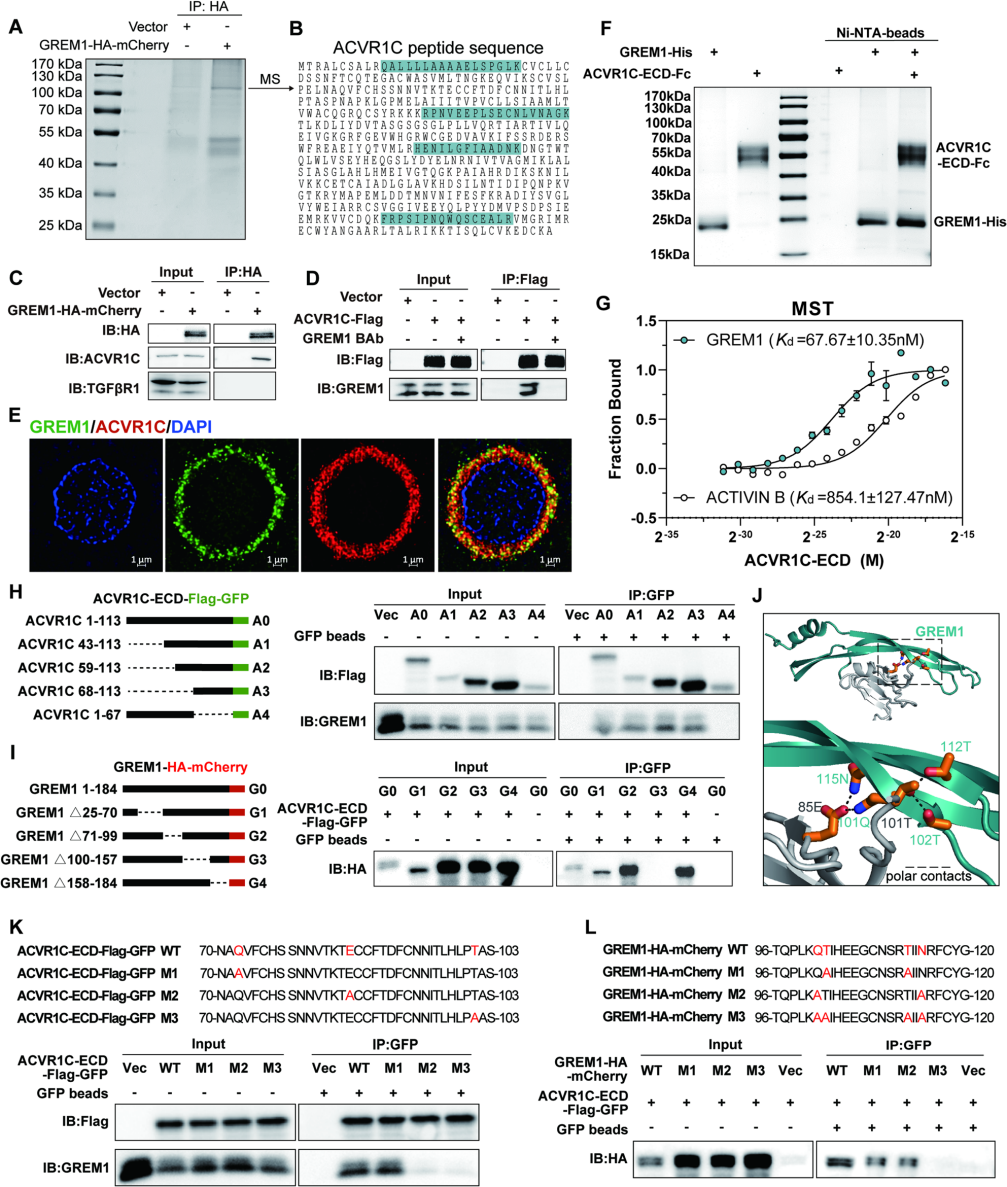
ACVR1C is a novel GREM1 receptor in CRC. **A**, Proteins extracted from HCT116 cells transfected with HA-mCherry-tagged GREM1 were incubated with magnetic beads conjugated to an anti-HA antibody. Bound proteins were eluted and visualized by Coomassie Brilliant Blue staining. A protein band of ~ 110 kDa was submitted for mass spectrometry (MS). **B**, The full amino-acid sequence of human ACVR1C. The sequences in blue are the tryptic peptides identified by MS. **C**, GREM1 co-immunoprecipitates with ACVR1C in HCT116 cells transfected with HA-mCherry-tagged GREM1. The bound proteins were immunoprecipitated with an anti-HA antibody and blotted by anti-HA, anti-ACVR1C, and anti-TGFβR1 antibodies. **D**, ACVR1C co-immunoprecipitates with GREM1 in HCT116 cells transfected with Flag-tagged ACVR1C. The bound proteins were immunoprecipitated with an anti-Flag antibody and blotted by an anti-GREM1 antibody. **E**, Confocal microscopy images of GREM1 and ACVR1C in SW480 cells. Scale bars, 1 μm. **F**, Interaction between purified GREM1 and ACVR1C-ECD proteins as demonstrated by pull-down experiments. **G**, Increasing concentrations of recombinant ACVR1C-ECD-Fc protein (0–27.5 µM) were incubated with red-fluorescently labeled recombinant GREM1-His or ACTIVIN B-His (50 nM). MST was used to evaluate the binding of ACVR1C-ECD-Fc to GREM1-His or ACTIVIN B-His (n = 3 independent experiments). Data are presented as mean ± s.e.m. **H**, **I**, Diagrams of truncated ACVR1C (**H**, Left) and truncated GREM1 (**I**, Left), with corresponding co-immunoprecipitation results (Right) comparing truncation mutants with binding partners. **J**, Molecular docking of GREM1 and ACVR1C-ECD simulated by HDOCK. Docking module highlighting key amino acid residues in the binding pocket between GREM1 and ACVR1C. **K**, Schematic of ACVR1C mutations (point mutations highlighted in red, Upper). Co-immunoprecipitation of ACVR1C and GREM1 is impaired by the AA_85_ mutant (M2) or the AA_101_ mutant (M3) of ACVR1C (Lower). **L**, Schematic of GREM1 mutations (point mutations highlighted in red, Upper). Co-immunoprecipitation of ACVR1C and GREM1 is impaired by the AA_101/102/112/115_ mutant (M3) of GREM1 (Lower)

The observed stage-dependent shift to epithelial GREM1 expression suggested that a stromal-derived signal triggers a transition to tumor-autonomous signaling. We therefore hypothesized that CRC cells express a cognate receptor for stromal GREM1, and that ligation of this receptor initiates a signaling cascade that both induces *GREM1* transcription in tumor cells and drives metastatic progression. To identify potential GREM1 receptors, we overexpressed HA-tagged GREM1 in the human CRC cell line HCT116 (Fig. 2A). Mass spectrometry analysis of proteins pulled down using anti-HA beads identified ACVR1C, a member of the TGFβ superfamily, as a potential GREM1 receptor (Figs. 2B and S3A).

To validate this interaction, we performed co-immunoprecipitation (co-IP) assays using HA-tagged GREM1 in HCT116 cells. Immunoblotting revealed that GREM1 interacted with ACVR1C specifically, while no such interaction was detected with other members of the TGFβ superfamily such as TGFβ receptor type I (TGFβR1) (Fig. 2C). Similarly, co-IP assays using Flag-tagged ACVR1C confirmed the interaction with GREM1, which was abolished by a GREM1-blocking antibody (BAb) (Fig. 2D). Confocal microscopy revealed the co-localization of ACVR1C with GREM1 in SW480 CRC cells (Fig. 2E). We next examined whether a direct interaction exists between GREM1 and ACVR1C. We found that Fc-tagged ACVR1C extracellular domain (ACVR1C-ECD, AA_1–113_) and His-tagged full-length GREM1 were pulled down together, demonstrating a direct physical association between ACVR1C and GREM1 (Fig. 2F). Further analysis of the binding affinity of GREM1 for ACVR1C using microscale thermophoresis (MST) revealed that ACVR1C-ECD exhibited a 12.6-fold higher affinity for GREM1 (*K*_d_ = 67.67 ± 10.35 nM) than that for ACTIVIN B (*K*_d_ = 854.1 ± 127.47 nM), a known ligand of ACVR1C [23] (Fig. 2G). To further delineate the explicit interaction mode of GREM1 and ACVR1C, we constructed truncated GREM1 and ACVR1C-ECD. Co-IP assays showed that deletion of amino acids 100–157 (AA_100–157_) in GREM1 or 68–113 (AA_68–113_) in ACVR1C-ECD effectively abolished their interaction in HCT116 cells (Figs. 2H, I). Based on these findings, we aimed to identify key residues mediating the interaction between GREM1 and ACVR1C. The HDOCK platform (https://dock.phys.hust.edu.cn/) was utilized to simulate potential docking modalities between GREM1 (https://csb.org/ PDB ID: 5AEJ) [24] and ACVR1C (AlphaFold2,https://alphafold.ebi.ac.uk/;UniProt ID: Q8NER5) based on their structures. Residues Q101/T102/T112/N115 in GREM1 and Q72/E85/T101 in ACVR1C were predicted to be essential for binding (Fig. 2J). To validate, we performed site-directed mutagenesis of the predicted residues to assess binding. Notably, the Q101A/T102A/T112A/N115A quadruple mutation in GREM1, or single mutations E85A or T101A in ACVR1C, significantly abrogated the GREM1–ACVR1C interaction (Figs. 2K, L). Further, we found no detectable interaction between recombinant GREM1 and the ACVR1C-ECD double mutant (E85A/T101A) by assessing their binding affinity using MST (Figure S3B). These data suggest that Q101/T102/T112/N115 in GREM1, and E85/T101 in ACVR1C are key residues mediating their interaction. Clinically, IHC and scRNA-seq revealed that ACVR1C is expressed in tumor cells, with markedly elevated levels in stage IV CRC (Figures S3C–E). Notably, high ACVR1C expression correlates with poor prognosis in stage IV CRC patients, supporting ACVR1C’s tumor-promoting role and consistent with the clinical significance of GREM1 (Figure S3F). Taken together, these results demonstrate that ACVR1C is a novel receptor of GREM1 in CRC.

### Secretory GREM1 induces EMT via the ACVR1C–SMAD2/3 pathway but not TGFβR/BMPR pathways

**Fig. 3:**
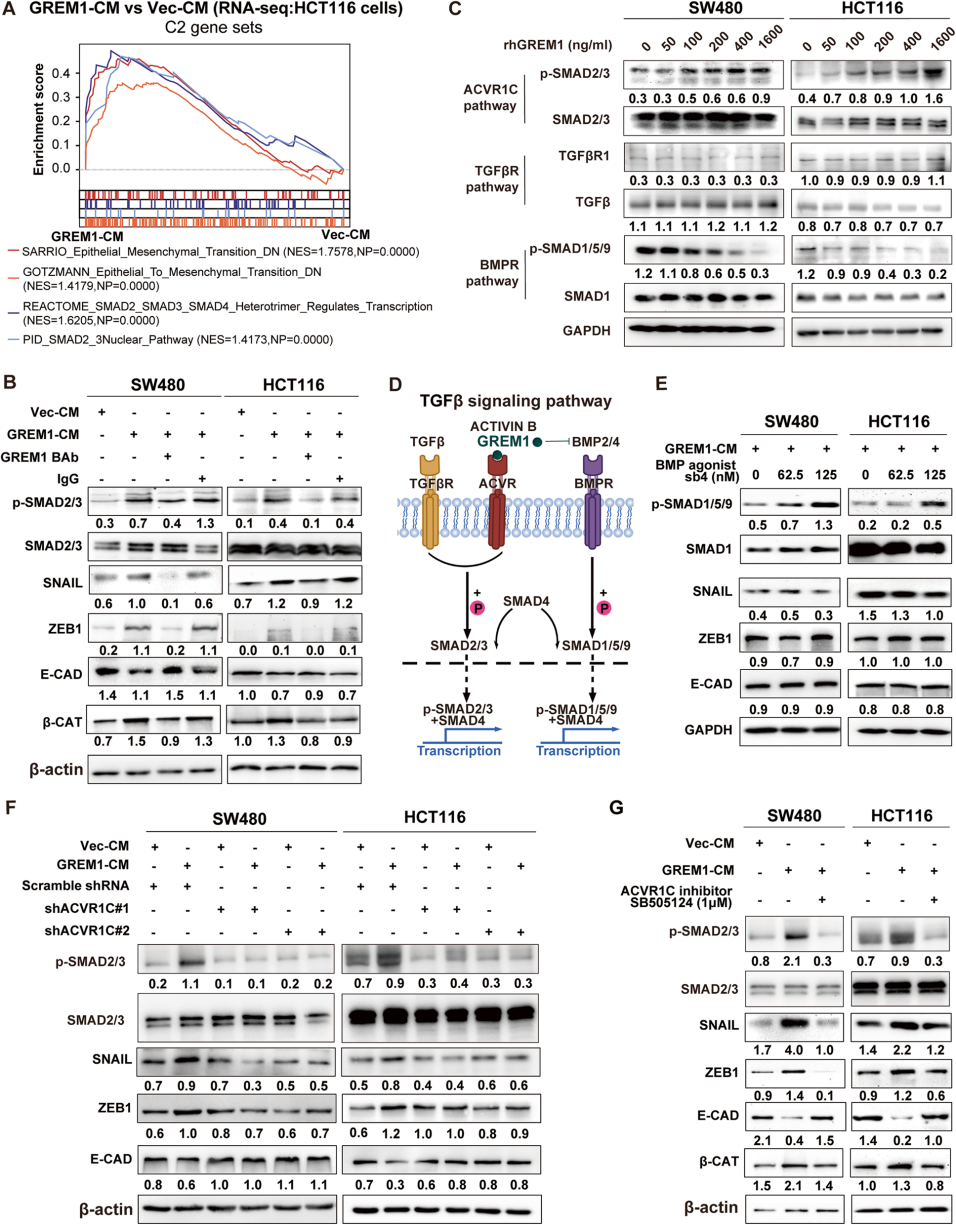
Secretory GREM1 binding of ACVR1C induces EMT via SMAD2/3 pathway. **A**, RNA sequencing was performed on HCT116 cells treated with GREM1 conditioned medium (GREM1-CM) or vector control conditioned medium (Vec-CM), followed by GSEA of the C2 gene sets. NES, normalized enrichment score; all *P* values equal to 0. **B**, Activation levels of the ACVR1C pathway markers (p-SMAD2/3 and SMAD2/3), as well as expression of the epithelial marker (E-CAD) and the mesenchymal markers (SNAIL, ZEB1, and β-CAT) were compared by immunoblotting analysis in SW480 and HCT116 cells treated with Vec-CM, GREM1-CM, GREM1-CM + GREM1 BAb, or GREM1-CM + IgG. **C**, Immunoblotting was performed to assess the ACVR1C pathway markers (p-SMAD2/3 and SMAD2/3), the BMPR pathway markers (p-SMAD1/5/9 and SMAD1), and the TGFβR pathway markers (TGFβR1 and TGFβ) from the lysates of HCT116 or SW480 cells with a concentration gradient of rhGREM1 treatment. **D**, Schematic representation of the TGFβ signaling pathway, highlighting GREM1-induced activation of ACVR1C and inhibition of BMPR signaling. **E**, Immunoblotting was performed to assess the BMPR pathway markers (p-SMAD1/5/9 and SMAD1) and expression of the epithelial marker (E-CAD) and the mesenchymal markers (SNAIL and ZEB1) from the lysates of HCT116 or SW480 cells treated with GREM1-CM and different concentrations of a BMP agonist, sb4. **F**, SW480 and HCT116 cells were transfected with control shRNA (Scramble shRNA) or with one of two ACVR1C-targeting shRNAs (shACVR1C#1 and shACVR1C#2) and separately treated with Vec-CM or GREM1-CM. Activation levels of the ACVR1C pathway markers (p-SMAD2/3 and SMAD2/3) and expression of the epithelial marker (E-CAD) and the mesenchymal markers (SNAIL and ZEB1) were analyzed by immunoblotting. **G**, Activation levels of the ACVR1C pathway markers (p-SMAD2/3 and SMAD2/3) and expression of the epithelial marker (E-CAD) and the mesenchymal markers (SNAIL, ZEB1, and β-CAT) were compared by immunoblotting analysis in SW480 and HCT116 cells treated with Vec-CM, GREM1-CM, or GREM1-CM + SB505124 (1 µM). SB505124 is a small-molecule inhibitor that selectively blocks the type I TGFβ/Activin receptors-mediated SMAD2/3 phosphorylation. For p-SMAD2/3, SMAD2/3 was used as a control. For p-SMAD1/5/9, SMAD1 was used as a control. For other proteins, β-actin was used as a loading control

To explore whether GREM1 serves as a functional ligand for ACVR1C in CRC cells, we first generated GREM1-enriched conditioned medium (GREM1-CM) and vector control conditioned medium (Vec-CM) using HEK293 cells (Figures S4A–C). We then performed RNA-sequencing (RNA-seq) on HCT116 cells treated with GREM1-CM or Vec-CM. Gene Set Enrichment Analysis (GSEA) revealed that SMAD2/3 and EMT pathways were significantly enriched in CRC cells treated with GREM1-CM (Fig. 3A). ACVR1C is one of the receptors of the TGFβ superfamily, and it transduces signals primarily through the phosphorylation of SMAD2/3 (p-SMAD2/3) [18]. Since commercial antibody for phosphorylated ACVR1C is not available, detection of p-SMAD2/3 serves as an effective proxy to reflect ACVR1C–SMAD2/3 activation. To confirm the effect of secretory GREM1 on the ACVR1C–SMAD2/3 pathway and EMT in CRC cells, we performed immunoblotting and found that GREM1-CM significantly increased p-SMAD2/3 levels in HCT116 and SW480 cells, which was effectively blocked by a GREM1 BAb (Fig. 3B). Moreover, immunoblotting and RT–qPCR analyses also revealed that GREM1-CM induced significant downregulation of E-CADHERIN (E-CAD, encoded by *CDH1*) [25] and upregulation of mesenchymal markers, including SNAIL (encoded by *SNAI1*), ZEB1, and β-CAT [22, 26] in SW480 and HCT116 cells. The GREM1 BAb effectively blocked GREM1-CM-induced EMT activation (Figs. 3B and S4D, E). Considering that EMT serves as an effective mechanism through which tumor cells acquire stroma-like traits to promote invasion and metastasis, we tested whether blocking GREM1 could inhibit the invasive and migratory capacity of CRC cells. Indeed, in vitro scratch and transwell assays showed that GREM1-CM significantly enhanced migration and invasion abilities of HCT116 and SW480 cells. Remarkably, these effects were abolished by GREM1 BAb treatment (Figures S4F–I). These findings suggest that secretory GREM1 activates the ACVR1C–SMAD2/3 pathway and promotes EMT and subsequent cellular behavior, such as migration and invasion of CRC cells.

The TGFβ superfamily signals initiate through BMP receptors (BMPRs), ACVRs, and TGFβRs. Although both ACVRs and TGFβRs converge on the SMAD2/3 axis [18], and GREM1-CM robustly activated TGFβ superfamily signaling in HEK293 cells (Figure S4J), our co-IP analysis showed no direct interaction between GREM1 and TGFβR1 (Fig. 2C). To determine whether GREM1 activates SMAD2/3 via TGFβR1 or ACVR1C, we treated SW480 and HCT116 cells with increasing concentrations of recombinant human GREM1 (rhGREM1). This led to a dose-dependent increase in p-SMAD2/3 (Fig. 3C), suggesting activation of a SMAD2/3-coupled receptor. To exclude the possibility that GREM1 indirectly stimulates TGFβ signaling, we examined whether rhGREM1 alters the expression of TGFβ or TGFβR1 in CRC cells. No changes were observed across all doses tested (Fig. 3C), indicating that GREM1 does not upregulate endogenous TGFβ signaling components. Together, these data suggest that GREM1-induced SMAD2/3 activation is unlikely to be mediated by TGFβR1 and instead proceeds via the ACVR1C pathway (Fig. 3D).

Considering that GREM1 is a canonical antagonist of BMP and that BMPRs exert their function through phosphorylation of SMAD1/5/9 (p-SMAD1/5/9), we sought to determine whether GREM1 regulates EMT via BMPR superfamily pathways. As expected, rhGREM1 suppressed the p-SMAD1/5/9 (Fig. 3C), in keeping with the canonical role of GREM1 as a BMP inhibitor. However, rescuing BMP signaling with the specific agonist sb4, which exclusively increases p-SMAD1/5/9 without affecting p-SMAD2/3 [27], failed to reverse GREM1-induced EMT (Fig. 3E). This definitive exclusion of BMPR–SMAD1/5/9 involvement establishes that GREM1 promotes EMT independently of its classical role as a BMP inhibitor.

Subsequently, to examine whether GREM1 promotes EMT through activation of the ACVR1C–SMAD2/3 pathway, we either stably knocked down ACVR1C (shACVR1C) or inhibited SMAD2/3 phosphorylation using a small‑molecule inhibitor, SB505124 [28]. Our immunoblotting and RT–qPCR analyses revealed that shACVR1C or SB505124 significantly blocked GREM1-CM–induced p-SMAD2/3 activation and EMT, indicating that ACVR1C–SMAD2/3 activation is required for GREM1-driven EMT in SW480 and HCT116 CRC cells (Figs. 3F, G and S5A, B and S6A, B). In addition, GREM1-CM-induced invasion and migration of these CRC cells was abolished by shACVR1C or SB505124 (Figures S5C–G and S6C–F). Collectively, these findings demonstrate that GREM1 induces EMT, as well as subsequent migration and invasion, by activating the ACVR1C–SMAD2/3 pathway.

p-SMAD2/3 form a complex with SMAD4 that translocates into the nucleus and acts as a transcriptional regulator [29]. *SNAI1* has been identified as a transcriptional target of the SMAD2/3/4 complex in several cancers, including CRC [30–32]. To define the direct binding sites involved in this regulation in CRC cells, we queried the JASPAR database (http://jaspar.genereg.net/) and identified three candidate SMAD2/3/4 binding sites (–967, −787, and −186) within the *SNAI1* promoter (Figure S6G). Chromatin immunoprecipitation (ChIP) followed by qPCR confirmed specific binding of SMAD2/3/4 to the −787 site. Importantly, inhibition of ACVR1C with SB505124 significantly decreased this binding (Figure S6H). Overall, our data reveal that secretory GREM1 is a specific functional ligand that activates the ACVR1C–SMAD2/3–SNAIL signaling axis, thereby promoting EMT, invasion, and migration of CRC cells.

### Depletion of Grem1^+^ stromal cells inhibits EMT and metastasis of CRC in vivo

**Fig. 4:**
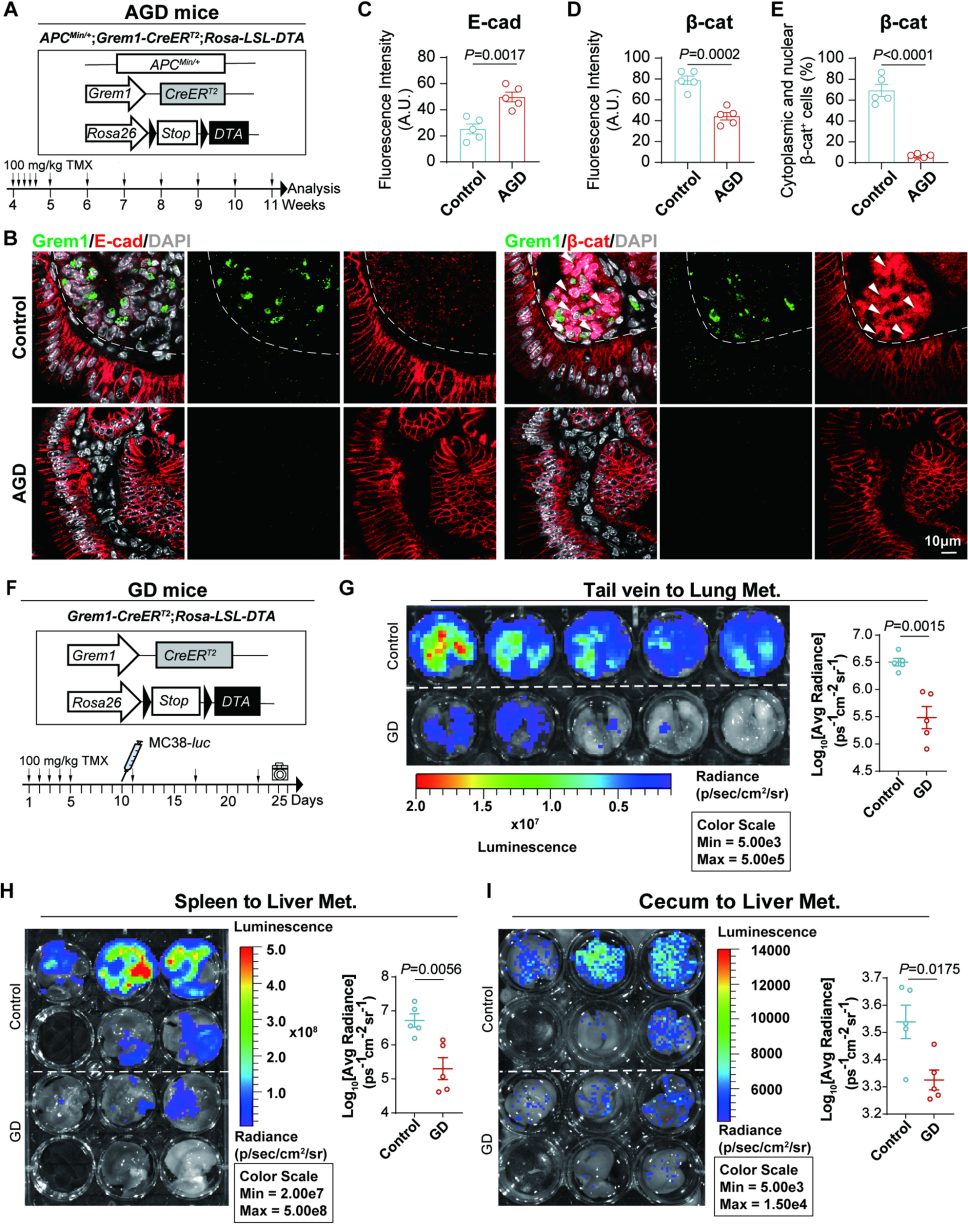
Depletion of Grem1^+^ stromal cells inhibits EMT and metastasis in vivo. **A**, Diagram of the AGD Mouse Model (*APC*^*Min/+*^;*Grem1-CreER*^*T2*^;*Rosa-LSL-DTA*) and the experimental approach. Grem1^+^ cells were selectively deleted through tamoxifen (TMX)-induced diphtheria toxin A (DTA) expression via Cre–loxP recombination. Mice received TMX (100 mg/kg) by oral gavage for five consecutive days starting at 4 weeks of age, followed by weekly TMX administration until analysis at 11 weeks. The black triangles indicate the loxP sites. **B**, Representative IF images showing staining for Grem1, E-cad, and β-cat in AGD mice or control mice (*APC*^*Min/+*^;*Grem1-CreER*^*T2*^ or *APC*^*Min/+*^;*Rosa-LSL-DTA*). The white irregular dashed boxes indicate regions infiltrated by Grem1^+^ stromal cells. Scale bars, 10 μm. **C**, **D**, Quantification of the fluorescence intensity of E-cad (**C**) and β-cat (**D**) in AGD and control mice shown in (**B**) (n = 5 mice per group). **E**, Quantification of the percentage of cells exhibiting cytoplasmic and nuclear translocation of β-cat under the conditions shown in (**B**) (n = 5 mice per group). **F**, Diagram of the GD mouse CRC model and the experimental approach. GD and control (*Grem1-CreER*^*T2*^ or *Rosa-LSL-DTA*) mice received TMX (100 mg/kg) by oral gavage for five consecutive days starting at 6 weeks of age, followed by weekly doses. At designated time points, MC38-luciferase (MC38-luc) cells were injected intravenously, intrasplenically, or into the cecal wall. Lung and liver metastases were imaged and quantified every five days using the IVIS Lumina Imaging System. The black triangles indicate the loxP sites. **G**–**I**, Representative images (Left) and quantification (Right) of metastatic burden in GD and control mice injected with MC38-luc cells, assessed by the IVIS Lumina Imaging System (n = 5 mice per group). Lung metastases following intravenous injection (**G**), liver metastases following intrasplenic injection (**H**), and liver metastases following cecum wall injection (**I**). Met., metastases. For **C**–**E** and **G**–**I**, data are presented as mean ± s.e.m. *P* values were calculated using two-tailed Student’s t-test

To validate the GREM1–ACVR1C axis-induced EMT and metastasis of CRC cells in vivo, we applied genetic and pharmacological strategies (Figure S6I). First, we evaluated the impact of stromal paracrine GREM1 on CRC in vivo. To block exogenous GREM1, we crossed *Grem1-CreER*^*T2*^;*Rosa-LSL-DTA* mice with *APC*^*Min/+*^ mice [33], a well-established model for CRC initiation, proliferation, and EMT that develops intestinal tumors by 10 weeks [34–36]. This cross generated AGD mice for TMX-induced depletion of GREM1^+^ stromal cells (Fig. 4A). Intriguingly, ablation of Grem1^*+*^ stromal cells for 6 weeks postnatally did not significantly alter the number or size of intestinal tumors in *APC*^*Min/+*^ mice (Figures S7A, B). Subsequently, we delved deeper into whether loss of paracrine GREM1 could restrain the malignant potential of intestinal tumor cells. In normal epithelia, β-CAT is tethered at the membrane by E-CAD [37]. During EMT, E-CAD loss enables β-CAT translocation to the nucleus [35, 38]. Consistent with this paradigm, tumor regions in *APC*^*Min/+*^ mice with GREM1^*+*^ stroma exhibited classic EMT features: a marked loss of E-CAD and robust β-CAT accumulation in the cytoplasm and nucleus (Fig. 4B, upper panels). Conversely, upon genetic depletion of GREM1^*+*^ stromal cells (AGD mice), epithelial integrity was preserved, with E-CAD intact at the membrane and β-CAT expression reduced and confined to membrane compartments (Fig. 4B, lower panels; Figs. 4C–E). These data indicate that stromal-derived Grem1 serves as a key extrinsic factor driving EMT in intestinal tumor cells. Next, to evaluate the impact of paracrine Grem1 on CRC metastasis, we injected luciferase-labeled murine rectal cancer cells (MC38-luc) into Grem1^*+*^ cell-depleted (GD) or control mice (Figures S7C, D). Cells were administered via the tail vein to induce lung metastasis, or into the spleen or cecum wall to induce liver metastasis [39] (Figures S7E–G). Strikingly, we observed a significant reduction in lung and liver metastases of CRC cells in GD mice compared with *Grem1-CreER*^*T2*^ or *Rosa-LSL-DTA* controls (Figs. 4F–I and S7H–J), suggesting that the stromal factor Grem1 is vital for CRC metastasis.

### Inhibition of ACVR1C–SMAD2/3 pathway inhibits GREM1-induced EMT and metastasis of CRC in vivo

**Fig. 5:**
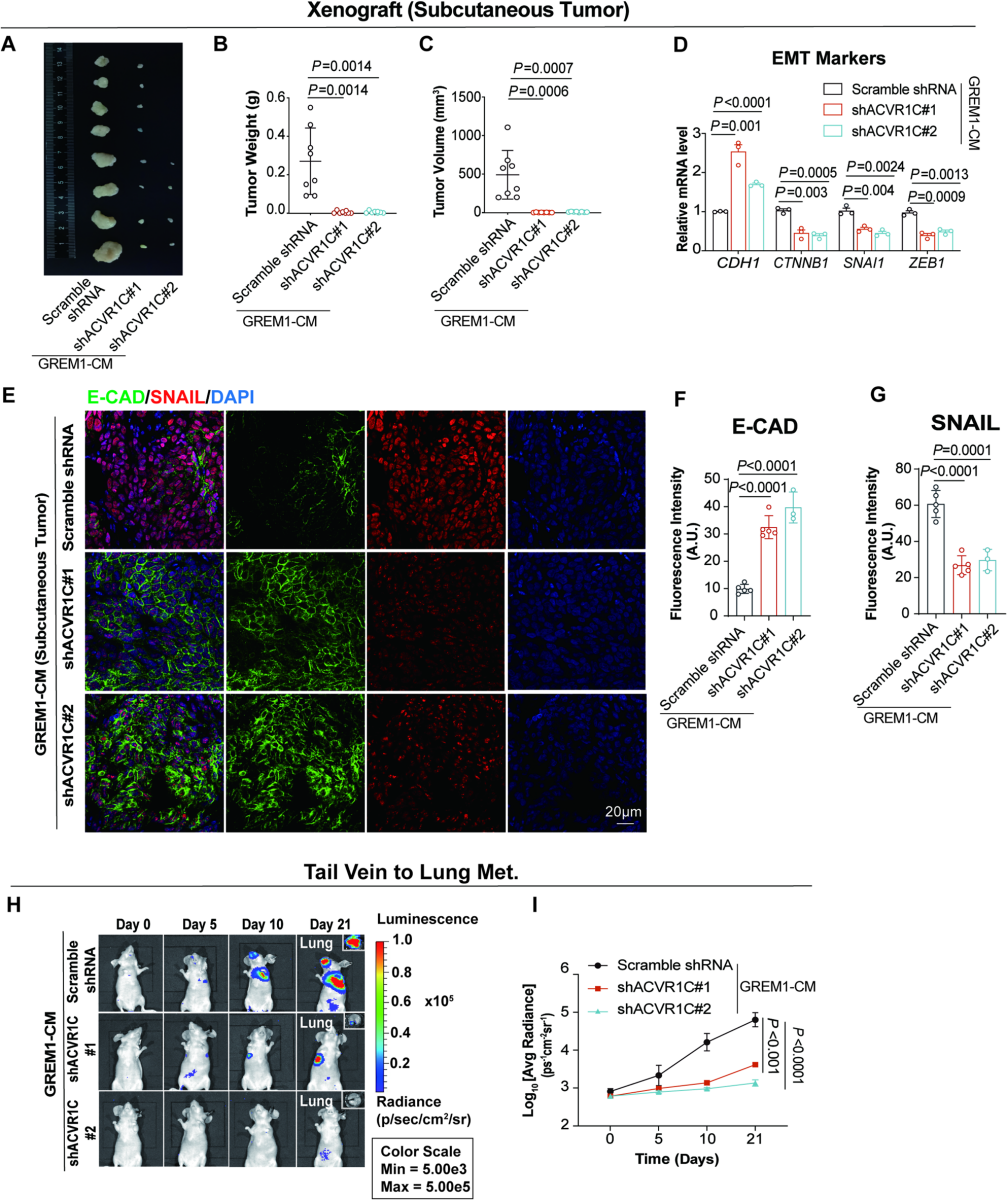
Knockdown of *ACVR1C* abolishes GREM1-CM-induced EMT and metastasis in CRC in vivo. **A**–**C**, Representative images (**A**), tumor weight (**B**), and tumor volume (**C**) of subcutaneous tumors from three groups of nude mice (n = 8 per group). Mice were subcutaneously injected with HCT116 cells transduced with scramble shRNA (control), or with shACVR1C#1 or shACVR1C#2, all pretreated with GREM1-CM. **D**, RT–qPCR analysis of mRNA levels of epithelial marker (*CDH1*) and mesenchymal markers (*CTNNB1*, *SNAI1,* and *ZEB1*) in subcutaneous tumors (n = 3 independent experiments). **E**–**G**, Representative IF images (**E**) and quantification (**F**, **G**) of E-CAD (green) and SNAIL (red) expression in subcutaneous tumors. Knockdown of *ACVR1C* reduced SNAIL expression and increased E-CAD expression. Fluorescence intensity was quantified in 3–5 mice per group. **H**, **I**, Representative IVIS images (**H**) and quantification (**I**) of lung metastases. Mice were injected intravenously with HCT116-luciferase (HCT116‐luc) cells, which were transduced with scramble shRNA or shACVR1C and treated with GREM1-CM. Bioluminescence signals from metastatic lesions were monitored over time using the IVIS Lumina Imaging System. Bioluminescence signals in the harvested lungs were visualized at day 21 post-tumor injection (upper right panel). For **B**–**D**, **F**, **G** and **I**, data are presented as mean ± s.e.m. *P* values were calculated using one-way ANOVA with Bonferroni’s multiple-comparison test

To determine the role of the ACVR1C–SMAD2/3 axis in GREM1-mediated EMT in vivo, we harnessed HCT116 cells carrying a luciferase reporter (HCT116-luc), which stably express either shACVR1C or a scramble shRNA. These cells were pretreated with GREM1-CM prior to their subcutaneous transplantation into nude mice. Notably, *ACVR1C* knockdown significantly inhibited subcutaneous tumor growth (Figs. 5A–C), accompanied by a significant increase in epithelial gene expression (e.g., *CDH1*) and a marked decrease in mesenchymal gene expression (e.g., *SNAI1*,* CTNNB1*, and *ZEB1*), as shown by RT–qPCR analysis (Fig. 5D). These findings were further corroborated by IF staining of EMT markers, including E-CAD and SNAIL (Figs. 5E–G). As a complementary pharmacological approach, we inhibited ACVR1C-mediated SMAD2/3 activation using SB505124. This treatment similarly suppressed tumor progression and EMT (Figures S8A–G), validating the essential role of the ACVR1C–SMAD2/3 pathway in GREM1-driven EMT. Importantly, to investigate the impact of the ACVR1C–SMAD2/3 axis on metastasis, we injected GREM1-CM-pre-treated HCT116-luc cells, stably expressing either shACVR1C or scramble shRNA, into the tail vein of nude mice. Remarkably, lung metastasis was profoundly suppressed in the *ACVR1C* knockdown group compared with controls (Figs. 5H, I). In parallel, we inoculated GREM1-CM-pre-treated HCT116-luc cells into the cecum wall of NOG (NOD/Shi-scid/IL-2Rγ) mice, a new generation of severely immunodeficient mice [40]. We found that SB505124 treatment resulted in a significant reduction in liver metastasis (Figures S8H, I). Collectively, these findings establish the ACVR1C–SMAD2/3 axis as a critical effector of stroma-derived GREM1, and reveal that its inhibition provides an effective strategy to counteract GREM1-induced EMT and metastasis in CRC in vivo.

### Stromal GREM1 confers tumor-cell signaling autonomy via the GREM1–ACVR1C axis in both primary and metastatic sites

**Fig. 6:**
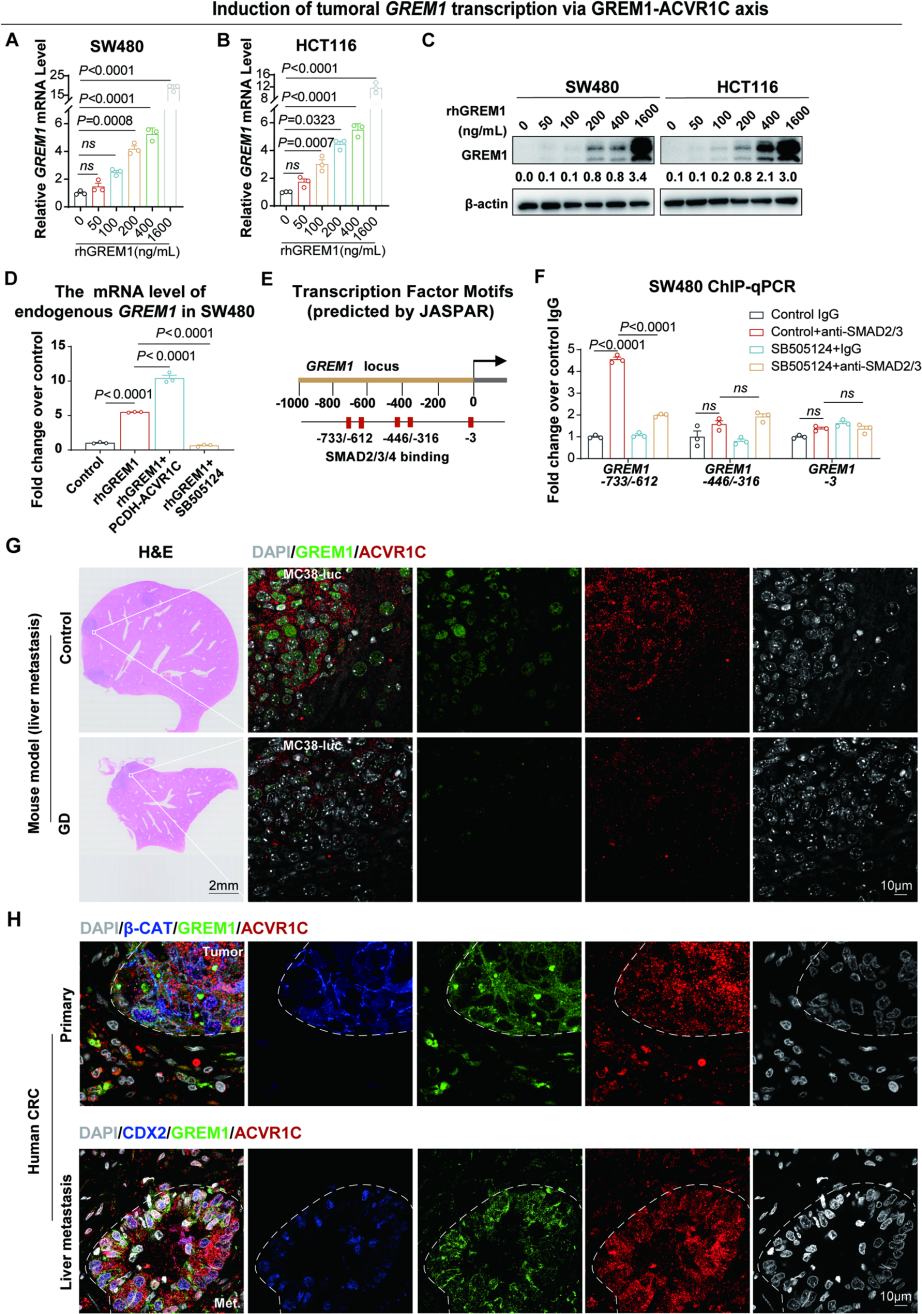
Exogenous stromal GREM1 confers tumor-cell signaling autonomy via the GREM1–ACVR1C axis in both primary and metastatic sites. **A**, **B**, RT–qPCR analysis of *GREM1* mRNA levels in SW480 (**A**) and HCT116 (**B**) cells treated with increasing concentrations of rhGREM1 (n = 3 biological replicates). **C**, Immunoblotting of GREM1 protein levels in SW480 and HCT116 cells treated with the same concentration gradient of rhGREM1 as in (**A**) and (**B**). **D**, RT–qPCR analysis of *GREM1* expression in SW480 cells (n = 3 biological replicates). **E**, SMAD2/3/4 binding motifs in the *GREM1* promoter, as predicted by the JASPAR database. Red boxes denote predicted binding sites upstream of the transcription start site (TSS). **F**, ChIP analysis of p-SMAD2/3 binding sites on the *GREM1* promoter in SW480 cells, treated as indicated. IgG control or SMAD2/3 antibodies were used for ChIP, and DNA enrichment was quantified by qPCR. DNA levels for each condition were normalized to the input, and the fold changes were calculated relative to the vehicle control (n = 3 independent experiments). **G**, H&E staining and IF analysis of liver metastatic lesions from MC38-luc tumor-bearing control and GD mice. Metastatic foci in control mice exhibit higher GREM1 and ACVR1C expression compared with those in GD mice. **H**, IF validation of GREM1 and ACVR1C expression in primary and liver metastatic CRC lesions. Representative IF staining of paired primary CRC (Upper panel) and liver metastasis (Lower panel) from the same patient. In primary tumors, β-CAT (blue) marks epithelial tumor cells, whereas in liver metastases, CDX2 (blue) identifies colorectal-origin metastatic cells. GREM1 (green) and ACVR1C (red) show co-localization within tumor epithelial regions in both primary and metastatic sites, indicating that tumor cells maintain detectable GREM1–ACVR1C signaling after dissemination to the liver. Nuclei were counterstained with DAPI (white). Met., Metastatic lesion. For **A**, **B**, **D**, and **F**, data are presented as mean ± s.e.m. *P* values were calculated using one-way ANOVA with Bonferroni’s multiple-comparison test. *ns*: not significant

The previous observation on the spatiotemporal shift of GREM1 from stromal to epithelial expression (Figs. 1A–C) prompted us to determine whether stromal GREM1 initiates tumor endogenous GREM1 expression. To test this, we treated SW480 and HCT116 cells with rhGREM1. RT–qPCR and immunoblotting analyses revealed a dose-dependent upregulation of tumor GREM1 expression in response to rhGREM1 stimulation (Figs. 6A–C), confirming that a paracrine GREM1 signal can trigger its autocrine expression in CRC cells. A pivotal question remained: why is this autocrine switch predominantly established in stage IV CRC, given the abundance of stromal GREM1 already in stage III (Fig. 1B, C, and S2D, S2E)? We reasoned that the initiation of this autocrine GREM1 might be gated by a limiting signaling component, specifically, the expression of its cognate receptor, acquired late in progression. Notably, the emergence of epithelial GREM1⁺ cells coincided with a marked upregulation of ACVR1C specifically in stage IV tumor epithelia (Figures S3D and S3E). We therefore postulated that elevated epithelial ACVR1C expression might be the crucial event enabling the transition from GREM1 paracrine stimulation to autocrine signaling. To definitively test whether GREM1 self-induction depends on the ACVR1C–SMAD2/3 pathway, we modulated this axis in SW480 cells by either overexpressing *ACVR1C* or inhibiting it with SB505124 following rhGREM1 treatment. Interestingly, *ACVR1C* overexpression further enhanced endogenous *GREM1* expression, whereas SB505124 treatment completely reversed the effect of rhGREM1 (Fig. 6D). To investigate whether SMAD2/3/4 act as transcription factors for *GREM1*, we utilized the JASPAR database and identified five candidate SMAD2/3/4 binding sites (−733, −612, −446, −316, and −3) in the *GREM1* promoter region (Fig. 6E). To validate the predicted results, we performed a ChIP–qPCR analysis, which revealed that SMAD2/3/4 bound the *GREM1* promoter at the −733 and −612 sites. Notably, SB505124 treatment significantly reduced this binding (Fig. 6F). These data demonstrate that exogenous GREM1 efficiently induces endogenous *GREM1* transcription in CRC cells via the ACVR1C–SMAD2/3 signaling pathway.

To further assess whether the tumor-cell signaling autonomy initiated by stromal GREM1 through activation of the GREM1–ACVR1C pathway can be sustained within metastatic sites, we conducted a cecum-to-liver metastasis experiment using MC38-luc cells (Fig. 4I). Metastatic foci were first identified by H&E staining and subsequently analyzed by IF staining to compare Grem1 and Acvr1c expression between control and GD mice, revealing robust expression of both molecules in metastatic lesions from control mice; in stark contrast, metastatic lesions in GD mice exhibited markedly reduced levels of both molecules (Fig. 6G). These findings indicate that stromal-derived Grem1 is essential for initiating and sustaining the Grem1–Acvr1c axis in metastatic tumor cells. In clinical samples, we further performed IF analysis on matched primary CRC tumors and liver metastases from the same patient. In the primary tumor, we co-stained with β-CAT to mark tumor epithelium, and in the liver metastasis, we used CDX2 (a transcription factor marking colorectal epithelial cells) [41] to definitively identify metastases of colorectal origin (Fig. 6H). This paired-tissue analysis provides evidence that the GREM1–ACVR1C axis remains active in CRC cells after they have colonized the liver microenvironment. To complement this, we turned to a publicly available scRNA-seq dataset of paired primary and metastatic CRC (GSE225857). This analysis revealed that in primary tumors, *GREM1* expression was present in both stromal cells and a subset of epithelial tumor cells. In liver metastases, it remained clearly detectable within a subpopulation of *EPCAM*^*+*^/*CDX2*^*+*^ metastatic tumor cells (Figure S9A). This supports our model that a subset of tumor cells maintains the ability to autonomously produce GREM1 during metastasis. *ACVR1C*, in contrast, was consistently expressed in tumor epithelial cells in both primary and metastatic sites, with negligible expression in fibroblasts (Figure S9B). This confirms that the receptor component is an intrinsic, obligate feature of CRC cells throughout the metastatic cascade. Together, these findings indicate that stromal GREM1 activates and sustains tumor-intrinsic GREM1–ACVR1C signaling, thereby enabling CRC cells to acquire signaling autonomy in both primary and metastatic sites.

### Tumoral GREM1 fuels EMT and metastasis independent of stromal signals

**Fig. 7:**
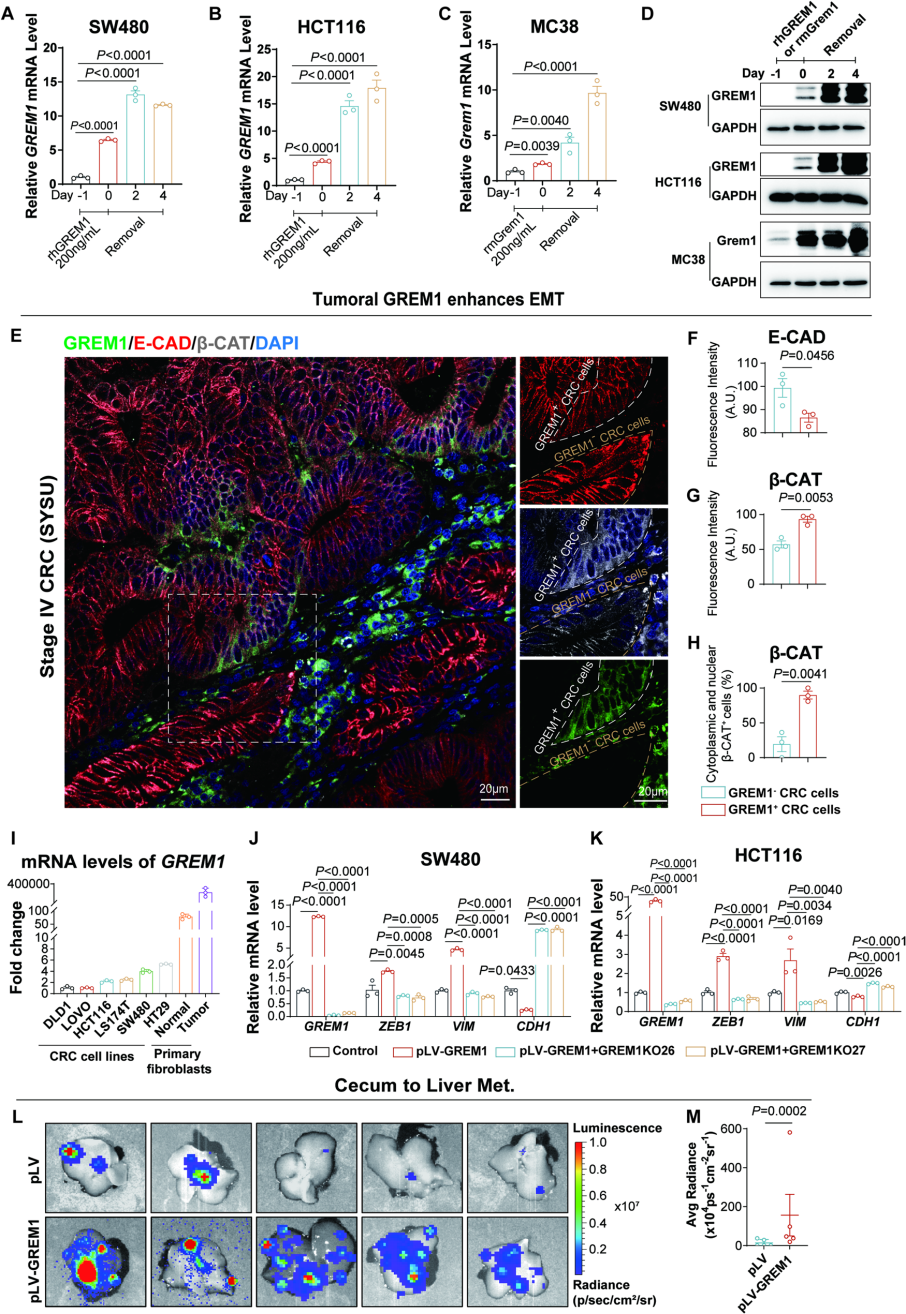
Autocrine GREM1 fuels EMT and metastasis independent of stromal signals. **A**–**C**, RT–qPCR analysis of *GREM1* mRNA levels in SW480 (**A**), HCT116 (**B**), and MC38 (**C**) cells treated with rhGREM1 or rmGrem1 (200 ng/mL) for 24 h, followed by removal of exogenous GREM1 and culture in GREM1-free medium for 2 and 4 days (Removal). Cells maintained elevated endogenous *GREM1* expression even after withdrawal of exogenous GREM1. **D**, Immunoblotting of endogenous GREM1 protein levels in SW480, HCT116, and MC38 cells treated with rhGREM1 or rmGrem1 for 24 h and subsequently cultured in GREM1-free medium for 2 and 4 days (Removal). GAPDH was used as a loading control. **E**, Representative IF images showing staining for GREM1, E-CAD, and β-CAT in stage IV human primary CRC tumors from SYSU. The white square-dashed box in the left image indicates the location of the corresponding region in the right image. The white irregular-dashed boxes mark the GREM1⁺ CRC cells, while the brown irregular-dashed boxes mark the GREM1^−^ CRC cells. **F**, **G**, Quantification of the fluorescence intensity of E-CAD (**F**) and β-CAT (**G**) in GREM1^+^ and GREM1^−^ CRC subpopulations shown in (**E**) (n = 3 patients). **H**, Quantification of the percentage of cells exhibiting cytoplasmic and nuclear translocation of β-CAT in the conditions shown in (**E**) (n = 3 patients). **I**, RT–qPCR analysis of *GREM1* mRNA expression levels in CRC cell lines (DLD1, LOVO, HCT116, LS174T, SW480, and HT29) and primary fibroblasts (normal fibroblasts and tumor derived-fibroblasts) derived from the gut of human CRC patients. **J**, **K**, RT–qPCR analysis of *GREM1*, the epithelial marker (*CDH1*), and the mesenchymal markers (*ZEB1* and *VIM*) in HCT116 cells infected with control (pLV) or GREM1-expressing (pLV-GREM1) lentiviruses, or in GREM1-expressing cells further transduced with one of two GREM1-targeting sgRNAs (GREM1KO26 and GREM1KO27) (n = 3 independent experiments). **L**, **M**, Representative IVIS images (**L**) and quantification (**M**) of liver metastases. NOG mice was injected in the cecum wall with HCT116-luc cells (pLV or pLV-GREM1). Liver metastases were imaged and quantified by the IVIS Lumina Imaging System (n = 5 mice per group). For **A**–**C**, **F**–**K** and **M**, data are presented as mean ± s.e.m. *P* values were calculated using one-way ANOVA with Bonferroni’s multiple-comparison test (**A**–**C**, **J**, **K**) and two-tailed Student’s t-test (**F**–**H**, **M**)

To determine whether tumor cells that have acquired signaling autonomy can sustain GREM1 expression in the absence of external stimulation, we performed a GREM1 withdrawal assay. CRC cells (SW480, HCT116, and MC38) were first pulsed with rhGREM1 or recombinant mouse GREM1 (rmGrem1) for 24 h to initiate the signaling response. The medium containing rhGREM1 or rmGrem1 was then thoroughly removed, replaced with fresh GREM1-free medium, and the cells were cultured for an additional 4 days. Remarkably, RT–qPCR and immunoblot analyses showed that the induced expression of endogenous GREM1 was not only maintained but in some cases further increased even after the withdrawal of exogenous GREM1 (Figs. 7A–D). This persistent, self-sustaining expression supports our conclusion that the initial rhGREM1 or rmGrem1 stimulation triggers a positive feedback loop of *GREM1* transcription within CRC cells.

Having established that exogenous GREM1 promotes EMT in CRC cells, we sought to determine whether endogenous GREM1 exerts a similar function. We began by delineating the bona fide correlation between epithelial GREM1 expression and EMT hallmarks using stage IV CRC clinical samples containing both GREM1^+^ and GREM1^−^ CRC cells. Remarkably, IF staining revealed that GREM1^+^ CRC cells exhibited a significant loss of E-CAD, along with increased β-CAT expression and its translocation to the cytoplasm and nucleus, compared to adjacent GREM1^−^ counterparts within the same tumor (Figs. 7E–H), suggesting that epithelial GREM1 expression contributes to EMT. We previously established that forced *GREM1* expression (pLV-GREM1) in CRC cells significantly enhanced their EMT and metastatic traits [13]. However, as endogenous GREM1 levels are inherently low in CRC cell lines compared to normal and cancer-associated fibroblasts (Fig. 7I), evaluating the necessity of GREM1, as well as modeling its therapeutic blockade, requires a detectable baseline. To this end, we utilized CRISPR/Cas9 to knock out *GREM1* in SW480 and HCT116 cells already stably expressing pLV-GREM1. RT–qPCR analysis confirmed that pLV-GREM1 induced a robust EMT profile, characterized by a marked decrease in *CDH1* and an increase in *SNAI1*,* VIM*, and *ZEB1*. Remarkably, subsequent *GREM1* knockout significantly reversed these changes, indicating that GREM1 is required to sustain the EMT program within CRC cells (Figs. 7J, K). These data demonstrate that endogenous GREM1 not only reinforces GREM1-driven signaling autonomy but also functions as a cell-intrinsic driver of EMT, providing a potential window for therapeutic intervention. To validate these findings *in vivo*, we first inoculated HCT116 cells carrying pLV-GREM1 into nude mice, which led to enhanced subcutaneous tumor growth compared to the pLV-empty vector control (Figures S10A–C). RT–qPCR analysis and IF staining of these tissues confirmed that tumor-autocrine GREM1 resulted in a molecular shift toward an EMT phenotype (Figures S10D–G). Furthermore, *GREM1*-overexpressing HCT116-luc cells showed a marked increase in lung and liver colonization following tail vein or cecal injection (Figs. 7L, M and S10H, I). Crucially, we tested whether tumor cell-derived Grem1 could independently sustain metastasis in the absence of a paracrine stromal supply. Using a spleen-to-liver metastasis model in GD mice, we observed that *Grem1*-overexpressing MC38-luc cells retained their metastatic capacity, performing comparably to those in control mice (Figures S10J–M). Collectively, these findings demonstrate that once CRC cells achieve GREM1-driven signaling autonomy, they become independent of stromal Grem1 inputs, maintaining their metastatic competence through a self-sustained, intrinsic program.

### Targeting the GREM1–ACVR1C interaction interface to inhibit metastasis of CRC in vivo

**Fig. 8:**
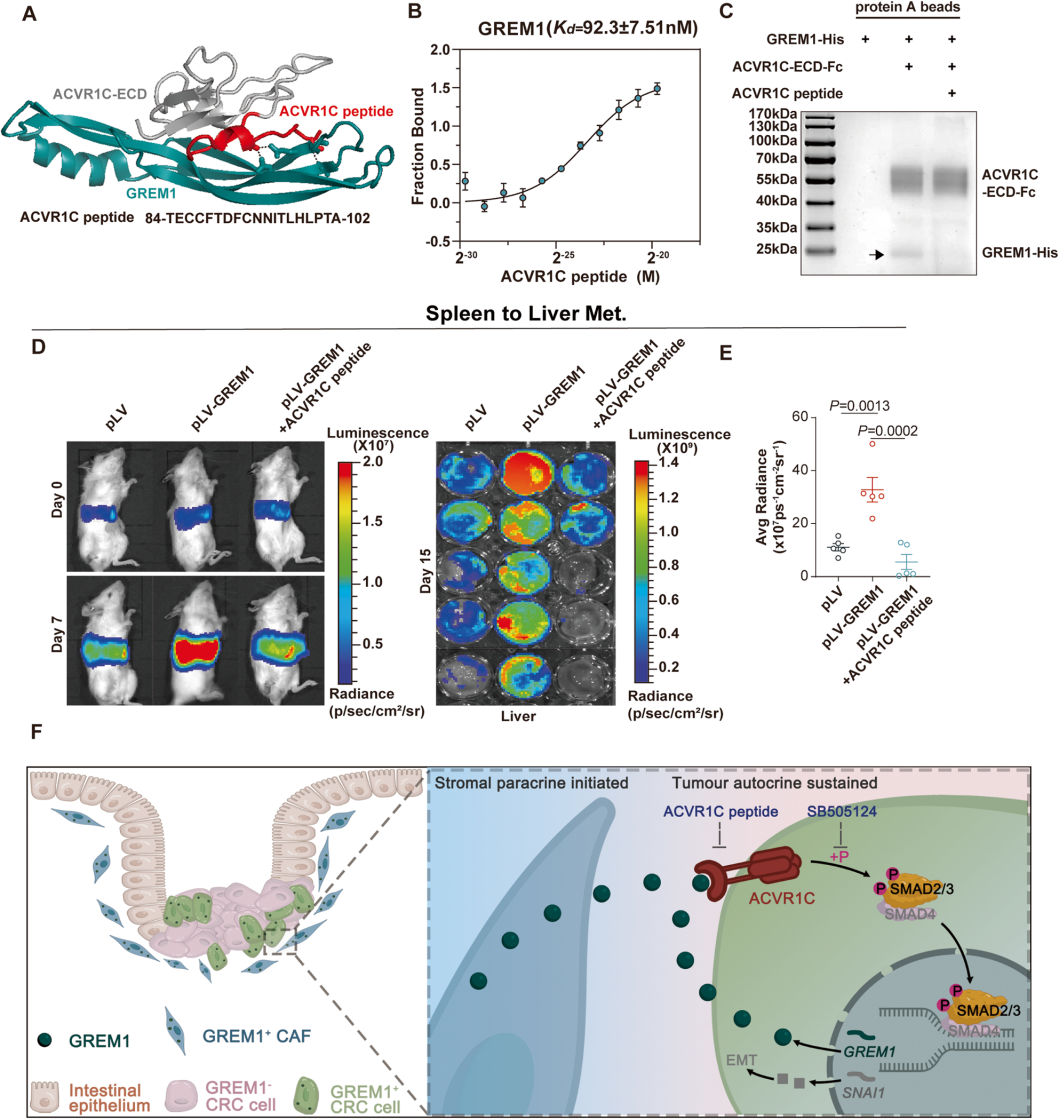
Targeting the GREM1–ACVR1C interaction interface to inhibit metastasis of CRC. **A**, Docking module of GREM1 and ACVR1C-ECD, highlighting key amino acid sequence between GREM1 and ACVR1C (ACVR1C peptide). **B**, Increasing concentrations of recombinant ACVR1C peptide (0–2.3 µM) were incubated with red-fluorescently labeled recombinant GREM1-His (50 nM). MST was used to evaluate ACVR1C peptide binding to GREM1-His (n = 3 independent experiments). **C**, Pull-down assays confirmed that the ACVR1C peptide blocked the interaction between GREM1 and ACVR1C-ECD. The black arrow indicates the GREM1-His band. **D**, **E**, Representative IVIS images (**D**) and quantification (**E**) of liver metastases. NOG mice were injected in the spleen with HCT116-luc cells (pLV or pLV-GREM1). Treatment was started 48 h after cell injection. ACVR1C peptide was administered intravenously every other day (10 mg/kg). Liver metastases were imaged and quantified by the IVIS Lumina Imaging System (n = 5 mice per group). **F**, A mechanistic model in which stromal paracrine GREM1 initiates an ACVR1C-dependent autocrine loop in tumor cells to orchestrate CRC metastasis. For **B** and **E**, data are presented as mean ± s.e.m. *P* values were calculated using one-way ANOVA with Bonferroni’s multiple-comparison test (**E**)

In clinical research, the molecular complexity and shared signaling of CRC limit the development of effective targeted therapy. While the TGFβ superfamily is a promising therapeutic axis [42], its intertwined and overarching roles in development and physiology make selective targeting difficult due to systemic toxicity [43, 44]. Targeting protein–protein interaction (PPI) interfaces is thought to further enhance specificity and reduce off-target effects [45]. Based on the principle of GREM1–ACVR1C interface disruption, we designed a peptide inhibitor derived from amino acid residues 84–102 (AA_84–102_) of ACVR1C (hereafter referred to as the ACVR1C peptide) (Fig. 8A). We further examined the binding affinity between GREM1 and the ACVR1C peptide using MST. We found that GREM1 exhibited an affinity for the ACVR1C peptide comparable to that for the ACVR1C-ECD (*K*_d_ = 92.30 ± 7.51 nM) (Fig. 8B). Pull-down assays showed that the introduction of ACVR1C peptide significantly blocked the GREM1–ACVR1C binding (Fig. 8C), suggesting that the peptide disrupts the GREM1–ACVR1C interaction by potently and competitively binding to GREM1. Further, we employed a spleen-to-liver metastasis model to evaluate the functional effect of the ACVR1C peptide in CRC metastasis. Notably, administration of the ACVR1C peptide effectively suppressed the increase in liver metastasis induced by tumor-specific *GREM1* overexpression (Figs. 8D, E), suggesting that our ACVR1C peptide significantly attenuated CRC progression by blocking the metastasis-promoting effects of tumor-autonomous autocrine GREM1–ACVR1C signaling, laying the foundation for the development of a novel class of peptide-based targeted therapies in CRC.

## Discussion

Whether tumor cells can internalize stromal paracrine signals and convert them into autocrine loops during metastasis has remained unclear. Here, we show that in advanced CRC, GREM1 undergoes a stromal-to-epithelial shunt and binds ACVR1C to activate SMAD2/3, inducing both *SNAI1* and *GREM1* expression. This establishes a self-amplifying autocrine circuit that drives EMT, conferring signaling autonomy and promoting metastasis (Fig. 8F). A functional peptide we designed for targeting the GREM1–ACVR1C interface effectively disrupts this loop and demonstrates therapeutic potential.

During embryonic development and tissue repair, intercellular communication is precisely regulated by paracrine and autocrine signaling pathways [46, 47]. Similarly, tumor cells exploit these dual signaling modes to enhance their survival and invasive capacity [48]. Moreover, during dissemination to distant sites, metastatic tumor cells have been observed to bring along components of the primary tumor stroma, including CAFs, reflecting their persistent reliance on microenvironmental support [49, 50]. Sporn et al. first proposed that a potential mechanism of malignant transformation is the autocrine production of growth factors to which the cell itself can respond, a process that may originate from the reactivation of autocrine strategies employed during early embryonic development, enabling cells to survive even in the absence of external support [51, 52]. Hanahan et al. suggested that paracrine signals from the stroma might trigger the emergence of signaling autonomy in tumor cells [53]. While neuron-secreted NLGN3 has been reported to upregulate NLGN3 expression in glioma cells, it has not been demonstrated whether this process evolves into a self-sustaining autocrine loop [54]. Thus, whether tumor cells can transition from paracrine induction to a self-sustaining autocrine circuit, remains unresolved. In this study, we show that during CRC metastasis, GREM1 expression undergoes a stromal-to-epithelial shunt, establishing an autocrine signaling mode initiated by paracrine cues. This transition suggests that metastatic cells acquire signaling autonomy by reactivating developmental autocrine programs, enabling them to survive and disseminate in distant organs with reduced microenvironmental support. Such a shift from “dependence on soil” to “self-construction of soil” reflects the remarkable adaptability of tumor cells.

While traditionally categorized as a BMP antagonist, portraying GREM1 solely as an inhibitor overlooks its diverse, sometimes paradoxical roles in various biological settings [55]. Accumulating evidence suggests that GREM1 possesses non-canonical, BMP-independent activities, engaging multiple receptors and activating diverse downstream signaling pathways. Previous studies have implicated EGFR [17] and VEGFR2 [16] as putative GREM1 targets, though their binding affinities have not been determined. Cheng et al. reported that GREM1 binds to FGFR1 with a dissociation constant of *K*_d_ = 10.6 nM [11]. In this study, we further identified ACVR1C [56] as a novel binding receptor for GREM1, with a dissociation constant of *K*_d_ = 67.67 ± 10.35 nM. Notably, this binding affinity is markedly higher than that of the canonical ACVR1C ligand ACTIVIN B (*K*_d_ = 854.1 ± 127.47 nM), representing a 12.6-fold increase. These results suggest that GREM1 engages ACVR1C with superior affinity, indicating its potential to act as a dominant ligand within this signaling pathway.

Unlike the mechanism described by Lan et al. in pancreatic cancer [10], where GREM1 inhibits EMT via classical BMP antagonism, our findings demonstrate that GREM1 binds ACVR1C and activates the SMAD2/3 axis independently of BMP signaling. This interaction initiates a positive autocrine feedback loop that promotes EMT and metastasis in CRC. These contrasting roles highlight the context-dependent plasticity of GREM1, which engages distinct signaling programs across tissue types to drive divergent outcomes.

Previous studies have established that the SMAD2/3 pathway transcriptionally promotes the expression of EMT factors such as *SNAI1* [30, 57], while its role and mechanism in regulating cell proliferation remain rather elusive [58, 59]. In keeping, our data consistently show that the primary function of the GREM1–ACVR1C–SMAD2/3 axis is to induce EMT and promote metastasis, but not to drive cell proliferation. Firstly, spatiotemporal expression analysis revealed a clinical correlation with the strong upregulation of both GREM1 and ACVR1C specifically in stage IV metastatic tumors (Figs. 1C, S2E and S3C–E), indicating their specialized role in late-stage, disseminated disease. Secondly, functional evidence (*in vitro* and *in vivo*) showed that genetic or pharmacological inhibition of ACVR1C–SMAD2/3 signaling almost completely abolished the GREM1-induced EMT and metastasis (Figs. 3F and G and 5D–G, S5A–G, S6A–F, and S8D–G). Albeit the preponderance of evidence reported in the present study supports the conclusion that the core function of the GREM1–ACVR1C–SMAD2/3 axis revolves around driving EMT and metastasis rather than proliferation, it should still be noted that the context-dependent biology of the individual signaling molecules, GREM1 and ACVR1C, must also be considered, alongside the GREM1–ACVR1C integrated axis.

In normal intestinal tissue, the Wnt/BMP counter-gradient spatially controls intestinal fate: high Wnt signaling in the crypt base maintains stemness and proliferation, while high BMP signaling in the villus drives differentiation, ensuring a strict balance between renewal and function [60]. In tumors, aberrant activation of GREM1 can relieve BMP-mediated suppression of stem-like proliferation, promoting the growth of HMPS [12], CRC [6], and other cancers [9, 61]. In such context, the pro-proliferative effect of GREM1 was also observed in our subcutaneous model (Figures S8A–C), which may be mediated through BMP inhibition. However, in the *APC*-mutant background, constitutive Wnt/β-catenin activation dominates tumor proliferation and masks GREM1’s proliferative role. The study by Davis et al. demonstrated that organoids derived from *APC*^*Min/+*^ adenomas could grow even without BMP antagonists, indicating that once Wnt is hyperactivated, BMP antagonism becomes dispensable [62]. Hence, in this genetic context, GREM1 signaling in our AGD model appeared dispensable for primary tumor formation (Figures S7A, B).

ACVR1C is known as an integrative node that receives signals from multiple ligands to activate not only canonical SMAD2/3 but also non-canonical pathways. For example, Nodal and Activin family ligands can activate AKT and ERK pathways via ACVR1C, thereby promoting tumor growth and invasion [63, 64]. Interestingly, ACVR1C itself may promote pancreatic cancer metastasis through a β-catenin/MMP axis [65]. Therefore, in the subcutaneous transplantation model, it is expected that *ACVR1C* knockdown not only disabled the multiple aforementioned ACVR1C-mediated downstream oncogenic signals but also ceased the GREM1 self-sustaining program (Fig. 6D), ultimately resulting in a complete inhibition of tumor growth (Figs. 5A–C).

The advent of cancer genomics and precision medicine has enabled targeted therapies for advanced CRC, with agents against VEGF, EGFR, BRAF V600E, and HER2 showing efficacy in selected patient subsets. However, issues like limited bioavailability, resistance, and narrow indications restrict their broader use [1]. Unlike mutation-specific targets, GREM1 is a widely expressed cytokine with sustained, context-dependent activity, making it a promising and potentially broadly applicable therapeutic target in CRC. Therapeutic targeting of GREM1 to date has focused largely on full neutralization strategies using monoclonal antibodies. Fully humanized monoclonal antibodies such as Ginisortamab and TST003 have advanced into phase I/II clinical trials [66, 67]. Although these antibodies have shown efficacy in preclinical models, the indispensable roles of GREM1 in physiological processes such as intestinal homeostasis and bone marrow hematopoiesis raise the risk of adverse effects from systemic inhibition, posing a major challenge to their clinical translation [68, 69]. In recent years, PPI have emerged as attractive therapeutic targets, with small peptides showing particular promise due to their high binding affinity and specificity [70]. In this study, we exploited the structural interface of the GREM1–ACVR1C interaction as a therapeutic entry point. Through structure-guided molecular design, we developed a high-affinity peptide capable of specifically disrupting this interaction, thereby markedly suppressing metastatic potential. Unlike conventional antibodies, our approach selectively targets the pathogenic GREM1–ACVR1C axis, highlighting the translational promise of rational PPI-targeted cancer therapies.

In summary, our findings reveal that the GREM1–ACVR1C axis acts as a key mediator of the stromal-to-epithelial shunt and the establishment of autonomous GREM1 signaling in CRC. By uncovering this pathway, we not only deepen our understanding of tumor self-sufficiency mechanisms, but also identify a functionally precise intervention point that opens new avenues for disrupting metastatic competence through targeted dismantling of self-reinforcing oncogenic circuits.

Despite these advances, our study has several limitations that warrant further investigation. First, the transcriptional regulation of *GREM1* and *ACVR1C* is likely to be highly complex. A previous study in gastric CAFs reported that loss of the repressive histone mark H3K27me3 de-represses *GREM1* transcription, highlighting the importance of epigenetic reprogramming in sustaining the GREM1^+^ CAF phenotype [71]. In the present study, our work defines a ligand-receptor signaling circuit as the principal mechanism for sustaining GREM1 and ACVR1C activity in metastatic CRC. However, epigenetic regulation, such as DNA methylation and histone modifications, may also contribute to the transcriptional control of *GREM1* and *ACVR1C*. These mechanisms would serve as important avenues for future research that complement our established signaling model.

Second, potential interactions between GREM1⁺ CAFs and ACVR1C⁺ immune cells warrant further exploration. Our analysis of public single-cell transcriptomic datasets showed that, in addition to CRC epithelial cells, moderate ACVR1C expression can also be detected in subsets of B cells and T cells (Figures S11A–C), suggesting a broader cellular distribution of this receptor within the TME. Zheng et al. reported that activins, members of the TGFβ superfamily, activate ACVR1C signaling to promote the differentiation of naïve CD4⁺ T cells into Foxp3⁺ induced regulatory T cells (iTregs), thereby suppressing anti-tumor immunity [72]. Furthermore, Kapoor et al. demonstrated that selective ablation of Grem1⁺ fibroblastic reticular cells in secondary lymphoid organs markedly reduces dendritic cell abundance and compromises T-cell immunity [73]. These findings raise the possibility that GREM1^+^ CAFs may promote tumor progression by activating ACVR1C signaling in immune cells through stromal GREM1. However, it is important to note that in our immunodeficient mouse models, genetic or pharmacological inhibition of ACVR1C using shACVR1C or SB505124 (Figs. 5H, I and S8H, I) markedly attenuated the metastatic phenotype induced by GREM1-CM. This suggests that even in the absence of ACVR1C⁺ immune cells, blocking tumor-cell–intrinsic ACVR1C signaling is sufficient to counteract the pro-metastatic effect of paracrine GREM1.

Finally, in early-stage CRC (stages I–III), the possible contribution of alternative GREM1 receptors beyond ACVR1C has not been fully elucidated. Our mechanistic evidence functionally and causally establishes ACVR1C as the dominant and indispensable receptor mediating GREM1 signaling particularly in stage IV metastatic CRC, where the GREM1–ACVR1C axis plays a central role in initiating EMT and driving metastatic dissemination. However, in earlier CRC stages where epithelial ACVR1C expression in tumor cells remains limited, we cannot exclude the possibility that GREM1 may exert some of its biological effects through other receptors, such as VEGFR2 [16], FGFR1 [11], or EGFR [17]. Analysis of public single-cell datasets revealed that while VEGFR2 and FGFR1 remain consistently low across tumor stages, EGFR expression progressively increases in tumor epithelial cells (Figures S11D–F). Thus, it would be of interest to investigate whether EGFR acts as a GREM1 receptor in early-stage CRC.

## Methods

### Cell lines

Human colon cancer cell lines (HCT116, SW480, DLD1, LOVO, LS174T, and HT29), mouse colon cancer cell line (MC38) and human embryonic kidney cell lines (HEK293 and its derivative 293T) were purchased from ATCC and tested negative for mycoplasma contamination. Colon cancer cells, HEK293 cells and 293T cells were cultured in RPMI1640 (HyClone, SH30809.01) and DMEM (SH30022.01), respectively, supplemented with 10% fetal bovine serum (Biological Industries, 04-001−1ACS) and 1% Penicillin/Streptomycin (Biological Industries, 03–031−1B) at 37 °C, 5% CO_2_.

### Patients and tissue samples

A total of 106 archived human colorectal cancer specimens were obtained from the colorectal cancer database and tissue bank of the First Affiliated Hospital of Sun Yat-sen University (SYSU). These tissues were collected from patients who underwent radical resection for colorectal cancer between 2008 and 2015, and followed up until December 2017. All patients had provided written informed consent, and the use of these samples was approved by the Institutional Review Board of the First Affiliated Hospital, SYSU. In addition, a matched pair of primary CRC tissue and corresponding liver metastasis was obtained from the tissue bank of Tongji Hospital, Tongji Medical College, Huazhong University of Science and Technology, with ethical approval granted by the hospital’s Medical Ethics Committee. Furthermore, a commercial human CRC tissue microarray containing primary tumor tissues from patients with stage I–III CRC (n = 93) was purchased from Shanghai Outdo Biotech Company (Shanghai, China), with ethics approval documented by the company.

### Conditioned medium (CM)

To investigate the effect of secreted GREM1 on CRC cells, an in vitro GREM1 secretion system was established using HEK293 cells. Stably GREM1-expressing cells (pcDNA3.1-GREM1) and empty-vector transfected cells (pcDNA3.1) were cultured in DMEM/F12 medium with 10% FBS until 40% confluence. After complete removal of the normal culture medium, HEK293-GREM1 and HEK293-Vec cells were continuously cultured in DMEM/F12 medium without FBS for 5 days before medium collection. GREM1 or Vector conditioned medium (GREM1-CM/Vec-CM) was then centrifuged at 1000 rpm for 30 min and the supernatant was collected for further study.

### Isolation of fibroblasts from normal and tumoral human intestinal tissues

Fibroblasts were isolated from normal and tumoral human intestinal tissues. Briefly, a cell dissociation buffer was prepared using DMEM supplemented with 10% FBS, 1% Penicillin/Streptomycin, collagenase type D (1 mg/mL, Roche, 11088866001), and DNase I (20 µg/mL, Roche, 10104159001). Tumor tissues were washed twice with DMEM or phosphate-buffered saline (PBS), transferred to a 100-mm culture dish containing 15 mL of cell dissociation buffer, and minced into fragments (< 1 mm³) using sterile razor blades. The tissue fragments were enzymatically digested at 37 °C for 30 min. Following digestion, the cell suspension was filtered through a 70 µm cell strainer to obtain a single-cell suspension. The cell suspension was centrifuged at 1000 rpm for 5 min, and the pellet was resuspended in DMEM. This step was repeated, and the final pellet was resuspended in DMEM supplemented with 10% FBS and 1% penicillin/streptomycin at 37 °C in a humidified incubator with 5% CO₂.

### Lentiviral plasmid construction, lentivirus production and infection

Human *GREM1* CDS (NM_013372.7) with a HA-mCherry tag or human *ACVR1C* CDS (NM_145259.3) with a Flag-GFP tag was cloned into a pCDH-CMV-MCS-EF1-puro vector. Truncated or point mutations of GREM1 or ACVR1C were cloned from entire GREM1- or ACVR1C-expressing plasmids by PCR. Human *GREM1* CDS (NM_013372.7) or mouse *Grem1* CDS (NM_011824.4) was cloned into the pLV-EF1a-IRES-Puro lentiviral vector. CRISPR-mediated gene knockout: The sequences targeting GREM1 were GREM1 KO27 (gRNA1: 5′ – GCAAATACCTGAAGCGAGAC − 3′) and GREM1 KO28 (g*RNA2*: 5′ – AAGCAGACCATCCACGAGGA − 3′). The Cas9 lentivirus and gRNA1/2 lentivirus were purchased from GenePharma. shRNA-mediated silencing: The human ACVR1C shRNA target sequences are listed as follows: shACVR1C#1 (5′ – CGGAGGAATTGTTGAGGAGTA − 3′); shACVR1C#2 (5′ – GCAACACCTCAACTCATCTTT − 3′). All inserts and vectors were purified from agarose gel using the FastPure ^®^ Gel DNA Extraction Mini Kit (Vazyme, DC301-01) and assembled with Gibson Assembly Master Mix [74] (NEB, E2611) according to the manufacturers’ protocols. All plasmids were verified by Sanger sequencing. HEK 293T cells were seeded at a density expected to reach 70–80% confluence at the time of transfection.

To produce lentivirus, plasmids mentioned above together with packaging plasmid (psPAX2) and envelope plasmid (pMD2.G) were mixed in a 3.9:2.1:1 ratio and transfected into the cells using polyethylenimine (PEI). After 48–72 h, supernatant containing lentivirus was collected, filtered, and either used immediately or stored at −80℃ for later applications.

SW480, HCT116, and MC38 tumor cells were infected with lentiviral particles in the presence of 5 µg/mL polybrene. To establish stable cell lines, these infected cells were selected with 1.25 µg/mL puromycin for 2 weeks.

### Animal experiments

The immunocompromised nude [75] and NOG [40] female mice (6 weeks old) were purchased from Guangzhou Vital River Laboratory Animal Technology Co., Ltd. *Grem1-CreER*^*T2*^ (stock no. 027039) [19], *Rosa-mTmG* (stock no. 007576) [76], and *Rosa-LSL-DTA* (stock no. 007900) mice [77] were obtained from the Jackson Laboratory. *APC*^*Min/+*^ mice were obtained from the Gempharmatech Co., Ltd (stock no. 002020) [33]. *Grem1-CreER*^*T2*^mice were crossed with *Rosa-mTmG* mice or *Rosa-LSL-DTA* mice to generate GR or GD mice, respectively. The Cre recombinase activity was induced by tamoxifen (TMX), allowing Grem1^+^ cells to express GFP in GR mice. GD mice were further crossed with *APC*^*Min/+*^ mice to generate AGD mice. AGD and control mice were administered with 100 mg/kg TMX through oral gavage at 4-week-old time, when tumor initiates. In AGD and its control mice, activation of Cre led to the expression of DTA (diphtheria toxin A chain), which removed the population of Grem1^+^ cells from the *APC*^*Min/+*^ mice. All animals were maintained at the Animal Experiment Center of Sun Yat-Sen University, and all procedures were approved by the Animal Care and Use Committee of Sun Yat-Sen University. Mice were randomized at the beginning of each experiment.

For tail vein-to-lung metastasis model, 5 × 10^5^ MC38-luc cells were resuspended in 100 µL of PBS and injected into the tail veins of GD mice or control mice (*Grem1-CreER*^*T2*^*/Rosa-LSL-DTA* mice) (n = 5 mice in each group); 1 × 10^6^ HCT116-luc cells, transduced with lentivirus carrying a control shRNA or two ACVR1C shRNAs or carrying a pLV or pLV-GREM1, were injected into the tail veins of nude mice (n = 5 mice in each group).

For spleen-to-liver metastasis model, 5 × 10^5^ MC38-luc cells or MC38-luc (pLV-Grem1) cells were resuspended in 50 µL of PBS and injected into the spleen of GD mice or control mice (*Grem1-CreER*^*T2*^*/Rosa-LSL-DTA* mice) (n = 5 mice in each group). 5 × 10^5^ HCT116-luc cells carrying a pLV or pLV-GREM1 were resuspended in 50 µL of PBS and intrasplenically injected into NOG mice (n = 5 mice in each group). ACVR1C peptide was administered via tail vein injection at a dose of 10 mg/kg once every other day.

For cecum-to-liver metastasis model, 1 × 10^6^ MC38-luc cells were resuspended in 50 µL of PBS and injected into the cecum of GD mice or control mice (*Grem1-CreER*^*T2*^*/Rosa-LSL-DTA* mice) (n = 6 mice in each group); 5 × 10^6^ HCT116-luc cells transduced with lentivirus carrying a pLV or pLV-GREM1 were injected into the cecum of NOG mice (n = 5 mice in each group). SB505124 was administered via intraperitoneal injection at a dose of 10 mg/kg once every other day. The metastases were examined every 5 days post injection using an IVIS Lumina Imaging System. Mice were euthanized between 2 and 6 weeks after injection. For subcutaneous transplantation, 1 × 10^6^ HCT116 cells, either unmodified or transduced with lentivirus carrying a control shRNA, two ACVR1C shRNAs, pLV, or pLV-GREM1, were subcutaneously injected into mice (n = 6–8 mice in each group). Mice were euthanized 4 weeks after injection. The tumor tissues were collected for further evaluation.

### Immunohistochemical (IHC) staining

Immunohistochemical staining of GREM1 (1:50, Biorbyt, orb10741), ACVR1C (1:50, Thermo, PA587475) and Ki67 (1:100, Servicebio, GB111499) was performed on primary tumor tissues. After dewaxing, hydration, and antigen retrieval, the rest of the experimental procedures were performed according to the instructions of the SP Immunohistochemistry Kit (ZSBIO, PV9000). Finally, after DAB staining, hematoxylin re-staining, and neutral resin sealing, the sections were observed under a microscope. Images were taken with a Slide Scanning Imaging System (SQS-1000, sqray). Quantification of positive staining was performed using Fiji (ImageJ).

### Immunofluorescence (IF) staining

Tissue was fixed in 4% paraformaldehyde (Thermo Scientific, I28800) for 24 h at 4 °C, washed with PBS, embedded in paraffin, and sectioned at 5 μm thickness. Antigen retrieval was performed using target retrieval solution, pH 9.0 in a pressure cooker for 15–20 min. Non-specific binding was then blocked with 10% normal donkey serum (Abcam, ab7475) and 0.3% Triton X-100 in PBS for 30 min at room temperature. Cells for IF were fixed with 4% paraformaldehyde for 20 min at room temperature, washed with PBS, and permeabilized with 0.2% Triton X-100 in PBS for 20 min. Cells were then blocked in PBS with 5% BSA for 30 min at room temperature. Subsequently, the samples were incubated with goat anti-GREM1 (3 µg/mL, R&D, AF956), mouse anti-β-Catenin (1:100, BD, 610154), rabbit anti-β-Catenin (1:100, Absea, RC-6352), rabbit anti-Vimentin (1:100, CST, 5741), rabbit anti-CD68 (1:100, CST, 26042), rabbit anti-FAP (1:50, Proteintech, 15384-1-AP), rabbit anti-α-SMA (1:100, Abcam, ab5694), rabbit anti-E-Cadherin (1:200, CST, 3195), rabbit anti-ACVR1C (1:50, Thermo, PA587475), rabbit anti-Snail (1:200, Abcam, ab224731) and mouse anti-CDX2 (1:200, Servicebio, GB151501) overnight at 4 °C. The tissues were incubated with Alexa-Fluor-conjugated secondary antibodies (Invitrogen) in PBS with 1% normal donkey serum for 1 h at room temperature. DAPI was then used for counterstaining the nuclei, and images were obtained by a laser scanning confocal microscope (LSM880, Zeiss).

### Analysis of scRNA-seq data

Single-cell RNA sequencing (scRNA-seq) data from the Gene Expression Omnibus (GEO) database colorectal cancer datasets (GSE200997 and GSE221575) were processed using the R ‘Seurat’ package (v4.4). Initial quality control involved rigorous filtering of low-quality cells: Cells expressing fewer than 200 genes or more than 10,000 genes were excluded, and cells with mitochondrial gene content exceeding 25% were discarded to remove potential apoptotic cells or debris. After quality control, a total of 34,675 high-quality cells were retained for downstream analysis. Gene expression matrices were normalized using the “LogNormalize” method implemented in the NormalizeData function, which scales feature counts per cell by total expression and multiplies by a scale factor (10,000), followed by natural log transformation. To identify biologically relevant features, the FindVariableFeatures function was employed to select the top 2,000 highly variable genes (HVGs) exhibiting the highest cell-to-cell variation. Dimensionality reduction was performed using principal component analysis (PCA) on scaled expression data of the identified HVGs. To address technical batch effects between samples and datasets, we applied multiple Canonical Correlation Analysis (CCA) as implemented in Seurat’s integration workflow. Cell clustering was performed using a graph-based approach: The FindNeighbors function constructed a shared nearest neighbor (SNN) graph based on the first 30 principal components, followed by the FindClusters function using the Louvain algorithm at a resolution of 0.8 to identify distinct cell subpopulations. Finally, non-linear dimensionality reduction was achieved through t-distributed Stochastic Neighbor Embedding (t-SNE) using the same principal components.

### Immunoprecipitation (IP)

HCT116 cells were transfected with the indicated plasmids and lysed in NP40 lysis buffer (Beyotime, P0013F) supplemented with protease inhibitor cocktail (Thermo, 78446). Lysates were incubated with the indicated Anti-Flag nanobody magarose beads (Ktsm-life, KTSM1338), Anti-HA nanobody magarose beads (Ktsm-life, KTSM1335) or Anti-GFP nanobody magarose beads (Ktsm-life, KTSM1334) overnight at 4 °C. The protein complex was washed four times with the NP40 lysis buffer, eluted with 1×loading buffer (Beyotime, P0015) by boiling for 5 min, followed by mass spectrometry and immunoblotting with the indicated antibodies.

### Mass spectrometry (MS) analysis

Proteins were separated by 10% SDS-PAGE and visualized using Coomassie Brilliant Blue staining before mass spectrometry analysis. The stained gel bands were excised (~ 1–2 mm), washed with MilliQ water, and destained using 25 mM NH₄HCO₃ and 50% acetonitrile (ACN) at 37 °C. The gel pieces were dehydrated with ACN, reduced with 10 mM dithiothreitol (DTT) in 25 mM NH₄HCO₃ at 37 °C for 1 h, and alkylated with 30 mM iodoacetamide (IAA) in 25 mM NH₄HCO₃ in the dark for 45 min. After sequential washing with MilliQ water and 50% ACN, the gel pieces were dehydrated with ACN and digested overnight at 37 °C with trypsin (20 ng/µL) in 25 mM NH₄HCO₃. Peptides were extracted using 60% ACN followed by pure ACN, pooled, lyophilized, resuspended in 0.1% formic acid (FA), and purified using ZipTip C18 before analysis. Mass spectrometry was performed using a Thermo Fisher Orbitrap HF-X coupled with an Easy-nLC 1200 system and a C18 column, employing a 90-min gradient of 5–35% ACN in 0.1% FA at a flow rate of 300 nL/min. MS1 scans were acquired at a resolution of 60,000 with an AGC target of 3 × 10⁶, a maximum injection time of 20 ms, and a scan range of m/z 350–1800. MS2 scans were performed at a resolution of 15,000 with an AGC target of 2 × 10⁵, a maximum injection time of 100 ms, TopN of 20, and a normalized collision energy (NCE) of 32. Raw MS data were analyzed using Proteome Discoverer 2.4, with protein identification performed against the SwissProt human database using trypsin specificity (allowing one missed cleavage site), cysteine alkylation with MMTS, a precursor mass tolerance of 10 ppm, a fragment mass tolerance of 0.02 Da, and a false discovery rate (FDR) threshold of < 1%.

### Immunoblotting (IB)

Protein was extracted from the cells with RIPA buffer (Beyotime, P0018) or NP40 lysis buffer (Beyotime, P0013F) and separated by SDS-PAGE, and transferred to polyvinylidene difluoride membranes. Primary antibodies against GREM1 (1:1,000, SinoBiological, 50016-R117), ACVR1C (1:1,000, Thermo, PA587475), Flag-tag (1:1,000, CST, 14793), HA-tag (1:1,000, CST, 3724), E-Cadherin (1:1,000, CST, 3195), β-Catenin (1:1,000, CST, 8480), ZEB1 (1:1,000, CST, 3396), Snail (1:1,000, CST, 3879), SMAD2/3 (1:1,000, CST, 8685), p-SMAD2/3 (1:1,000, CST, 8828), SMAD1 (1:1,000, CST, 6944), p-SMAD1/5/9 (1:1,000, CST, 13820), TGFβ (1:1,000, CST, 3709), TGFβR1 (0.3 µg/mL, R&D, AF3025), β-actin (1:5,000, Beyotime, AF0003) and GAPDH (1:5,000, Beyotime, AF0006) were used in this study. Peroxidase-conjugated secondary antibody (1:10,000, Cell Signaling Technology, 7074, 7076) was used and signal was visualized using an enhanced chemiluminescence assay (ECL, Thermo), according to the manufacturer’s protocol. Band intensity was quantified using Fiji (ImageJ) by grayscale analysis.

### Recombinant protein production and purification

Expi293F cells were transfected with a *pcDNA3.4-ACVR1C-ECD-Fc* and *pcDNA3.4-ACVR1C-ECD-Fc-double mutant (E85A/T101A)* expression vector to produce the target protein, which was subsequently purified using a Protein G column. Briefly, the coding sequence (CDS) of the human *ACVR1C* extracellular domain (*ACVR1C-ECD*, NM_145259.3, residues 1–113) fused to an Fc tag was cloned into the pcDNA3.4 vector. Expi293F cells were transfected with this construct, the supernatant was harvested 5 days post transfection.

The supernatant was first centrifuged at 1000 rpm for 20 min to remove cell debris, and the supernatant was further centrifuged at 8000 rpm for 30 min, followed by filtering with a 0.45 μm PES filter. The protein in the supernatant was then purified using a Protein G column equilibrated with binding buffer (0.15 M NaCl, 20 mM Na₂HPO₄, pH 7.0). The target protein was eluted with 0.1 M glycine (pH 2.5) and immediately neutralized with 1 M Tris-HCl (pH 8.5).

Subsequently, the protein buffer was exchanged into a 20 mM Tris-HCl (pH 7.5) system. To further purify the sample, it was centrifuged at 12,000 rpm and 4 °C for 10 min to remove impurities and precipitates. The clarified sample was then loaded onto an ion exchange column (HiTrap™ Capto™ Q ImpRes) equilibrated with binding buffer (20 mM Tris-HCl, pH 7.5). The ACVR1C-ECD-Fc protein, having an opposite charge to the resin, was bound to the column. Finally, the target protein was eluted with a linear gradient (0–100%) of elution buffer (20 mM Tris-HCl, 1 M NaCl, pH 7.5) over 6 column volumes.

### Protein pull-down assay

Protein pull-down assay was performed using purified recombinant human His-tagged GREM1 protein and recombinant human ACVR1C-ECD and Fc chimera protein. Protein was enriched by Pierce Protein A magnetic beads (MCE, HY-K0202) or Ni Sepharose 6 Fast Flow (GE, 17531801) following the manufacturer’s instructions. Pulled-down proteins were detected by Coomassie Brilliant Blue staining.

### MicroScale thermophoresis (MST)

MST was carried out on a Monolith NT.115 instrument (NanoTemper Technologies GmbH, MO-L011). To evaluate ACVR1C-ECD or ACVR1C peptide or ACVR1C-ECD-double mutant (E85A/T101A) binding to GREM1-His or ACTIVIN B-His, an increasing concentration of purified ACVR1C-ECD-Fc protein (0–27.5 µM) or ACVR1C peptide (0–2.3 µM) or ACVR1C-ECD-double mutant (0–27.5 µM) was incubated with 50 nM red-fluorescently labeled (NanoTemper Technologies GmbH, MO-L011) GREM1-His protein (R&D, 5190-GR) or ACTIVIN B-His protein (SinoBiological, 10814-H08H). Experiments were carried out in a PBS buffer pH 7.4 using premium capillaries (NanoTemper Technologies GmbH, MO-K025).

### Protein-protein interaction docking study

GREM1 (PDB: 5AEJ) was selected as the ligand and ACVR1C (PDB: AF-Q8NER5-F1) as the receptor for protein-protein docking. The HDOCK web service was used for docking with default parameters (http://hdock.phys.hust.edu.cn/). Key amino acid residues in the binding pocket between GREM1 and ACVR1C were further identified based on the docking module [78].

### RNA-seq and gene set enrichment analysis (GSEA)

Total RNA was extracted using Trizol reagent (Invitrogen, 15596026) and quantified with a NanoDrop spectrophotometer (Thermo Fisher Scientific). RNA integrity was assessed using an Agilent 2100 Bioanalyzer. mRNA was enriched using oligo(dT) magnetic beads, fragmented, and reverse-transcribed into cDNA. After adapter ligation and PCR amplification, libraries were sequenced on an Illumina platform, generating 150-bp paired-end reads. Raw reads were trimmed and aligned to the human reference genome (GRCh38) using STAR. Differential gene expression analysis was conducted using Limma, with significance thresholds set at |log_2_FoldChange| >1.5 and adjusted* P* value < 0.05. Gene set enrichment analysis (GSEA) was performed using the GSEA software (Broad Institute) with the MSigDB gene sets to identify enriched biological pathways, employing 1,000 permutations and FDR < 0.25 as the cutoff for significance.

### RT–qPCR

Total RNA was extracted using Trizol reagent (Invitrogen, 15596026). According to the instructions, cDNA was generated using the PrimeScript RT reagent Kit with gDNA Eraser (Accurate Biology, AG11706). The SYBR Green Premix Pro Taq HS qPCR Kit (Accurate Biology, AG11701) was then used to quantify mRNA expression according to the manufacturer’s instructions. All results were calculated using the 2^−ΔΔCt^ method. Primers used in the study are listed in Supplementary Table 1.

### ChIP

SW480 cells were starved in DMEM with 1% FCS overnight before treatment with vehicle, 10 µM SB505124 for 24 h. Cells were fixed in 1% paraformaldehyde for 10 min at RT for DNA-protein cross-linking, followed by quenching with glycine. Cross-linking chromatin was prepared using the SimpleChIP^®^ Enzymatic Chromatin IP Kit (CST, 9002) according to the manufacturer’s instructions. For immunoprecipitation, 10 µg chromatin was incubated with 10 µL anti-histone H3 rabbit IgG (CST, 14269, positive control), 2 µL normal Rabbit IgG (CST, 2729) or 5 µL anti-SMAD2/3 rabbit IgG (CST, 8685) at 4 °C overnight. 2% chromatin prior to immunoprecipitation was used as input. Chromatin-protein-antibody complex was captured by protein G magnetic beads, and chromatin was released by reversal of cross-links and purified using the SimpleChIP^®^ Enzymatic Chromatin IP Kit (CST, 9002) according to the manufacturer’s instructions. DNA was quantified by qPCR with primers targeting predicted SMAD2/3/4 binding regions on *GREM1* or *SNAI1* promoters. DNA levels were normalized to the input, and the fold-change of enrichment was calculated over the control. ChIP–qPCR primers are listed in Supplementary Table 2.

### Scratch assay

Cells were seeded into 6-well plates after centrifugation and digestion with 0.05% trypsin. When the cell density reached 90%, three vertical lines were scratched in each well with a 10 µL pipette tip and the floating cells were gently washed away with 1×PBS. Complete medium was added, and images of the scratch area were taken at 0 h. Three different fields of view were selected for each well. After photography, the medium was replaced with serum-free medium. Wound healing was documented at the same location after 24–48 h of incubation.

### Transwell invasion assay

 Cells (1 × 10^5^) were seeded in serum-free medium in the Matrigel-coated (Corning, 354480) transwell chambers (24-well insert, 8-µm pore size; BD Biosciences) for invasion experiments. The lower chamber was filled with RPMI1640 or DMEM containing 20% FBS. The migration of HCT116 and SW480 cells was measured in three random visual fields and quantified by microscopy after 48 h of incubation, followed by staining with DAPI or crystal violet. The invasive capacity of the cells was assessed using ImageJ software for quantification.

### Statistical analysis

All the statistical analyses were performed using GraphPad Prism 9, and error bars indicate s.e.m. Student’s t-test assuming equal variance and one-way analysis of variance for independent variance were used. Growth curves were generated using ANOVA for repeated measurement. *P* < 0.05 was considered significant. The number of independent experiments, the number of events and information about the statistical details and methods are indicated in the relevant figure legends.

## Supplementary Information

Supplementary Material 1.

Supplementary Material 2.

Supplementary Material 3.

Supplementary Material 4.

Supplementary Material 5.

## Data Availability

All datasets generated during this study are available in the Supplementary Materials accompanying this manuscript.
