# Supplementary material for: A paracrine-to-autocrine shunt of GREM1 fuels colorectal cancer metastasis via ACVR1C
Source: Mol Cancer. 2026 Jan 24;25:174. doi: 10.1186/s12943-025-02554-w (PMC13371181; doi:10.1186/s12943-025-02554-w)

**Raw images of the immunoblotting experiments**

**Figure 2:**


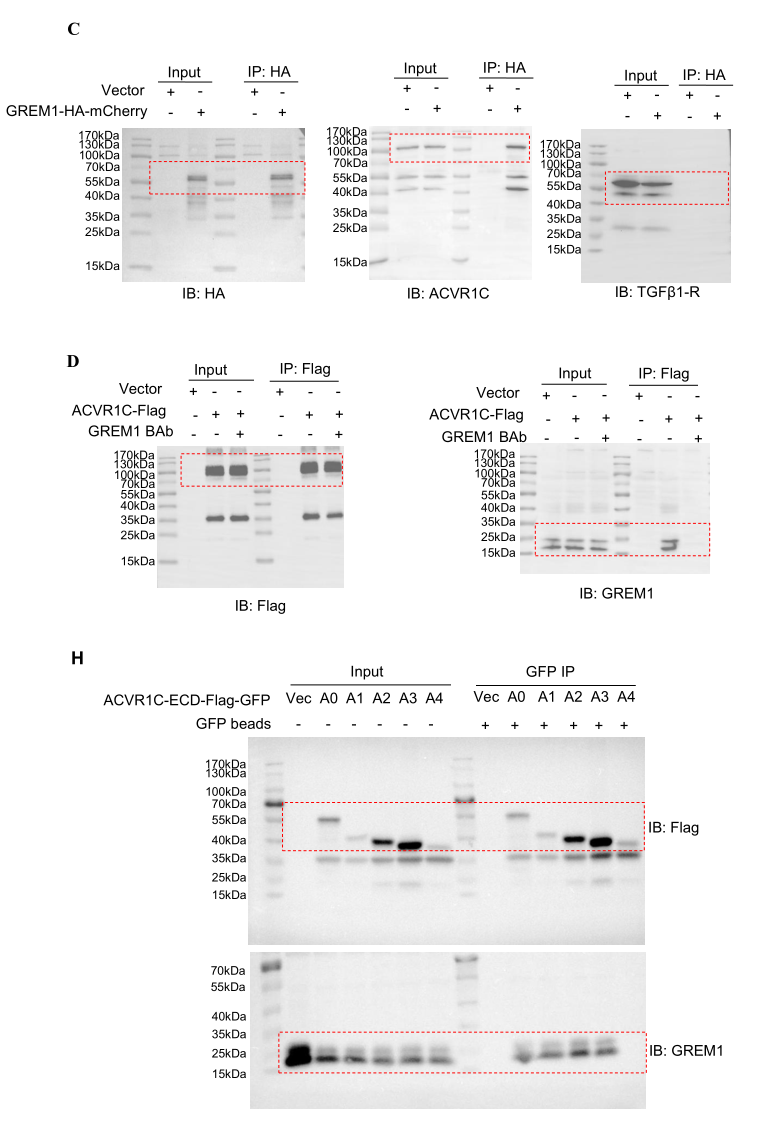


**Figure 2:**


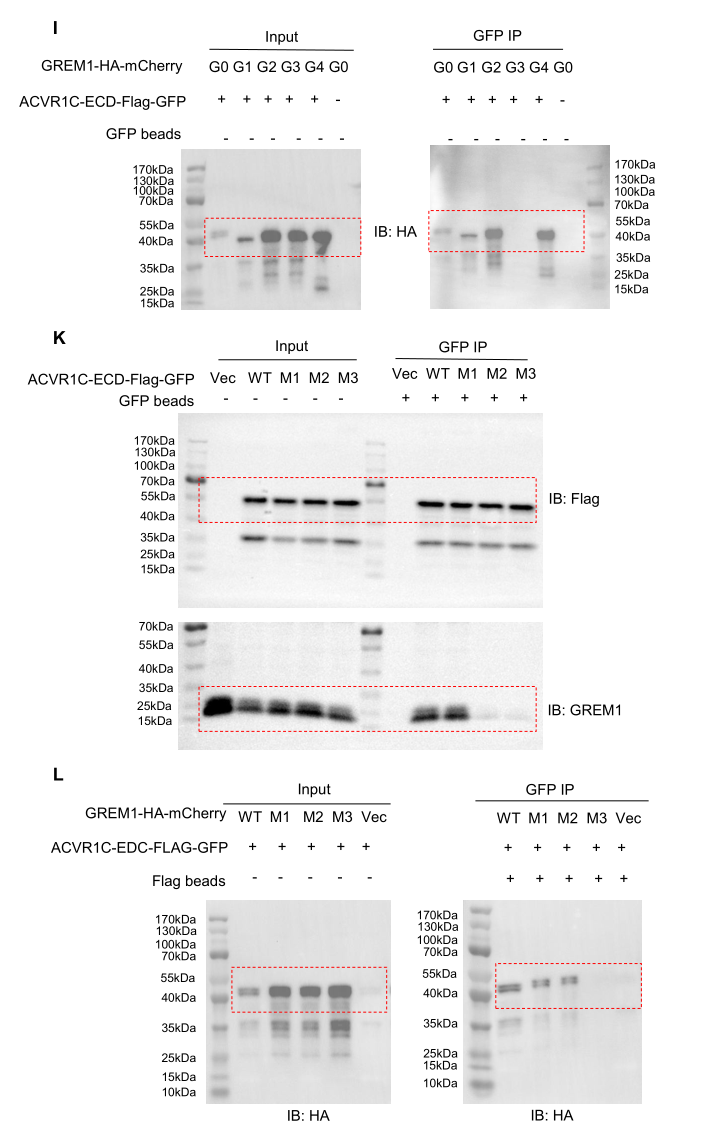


**Figure 3:**


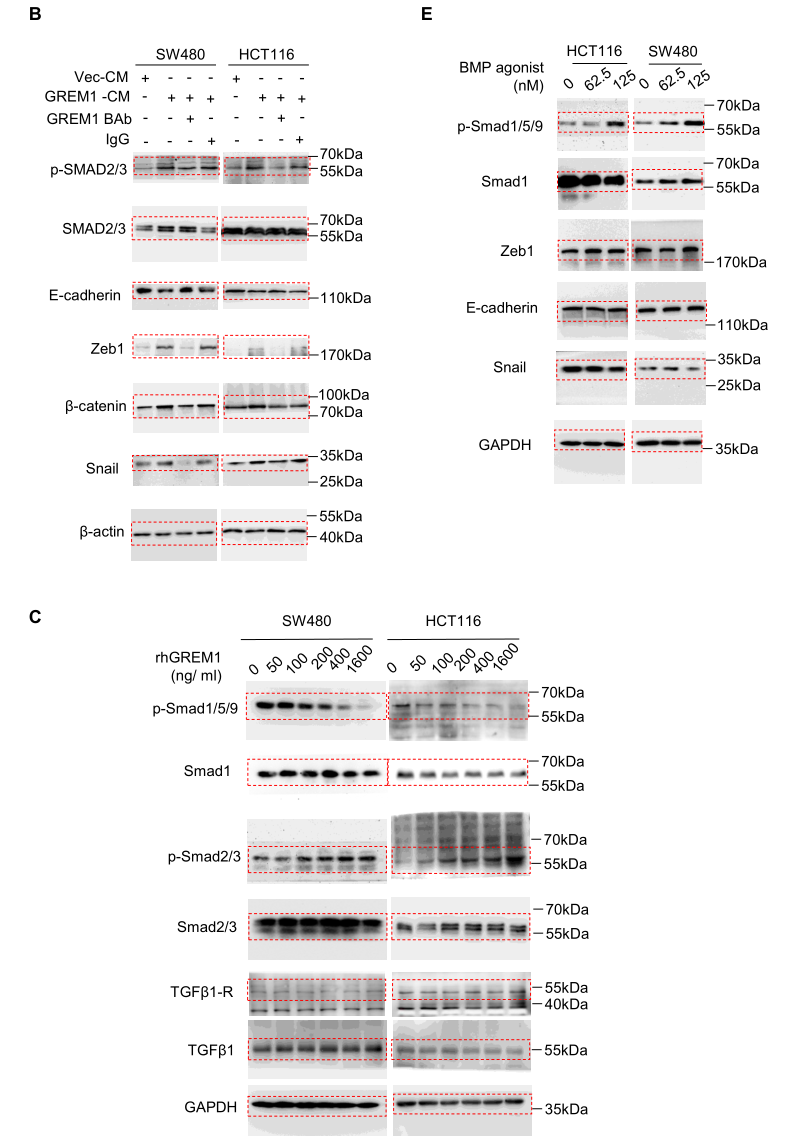


**Figure 3:**


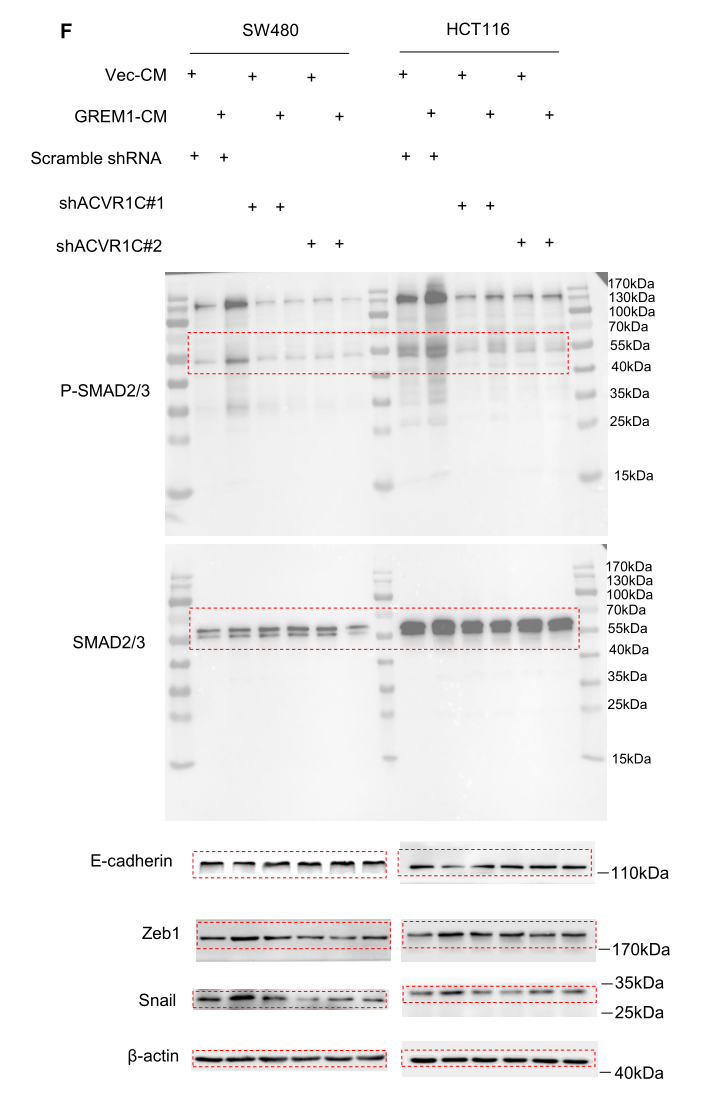


**Figure 3:**


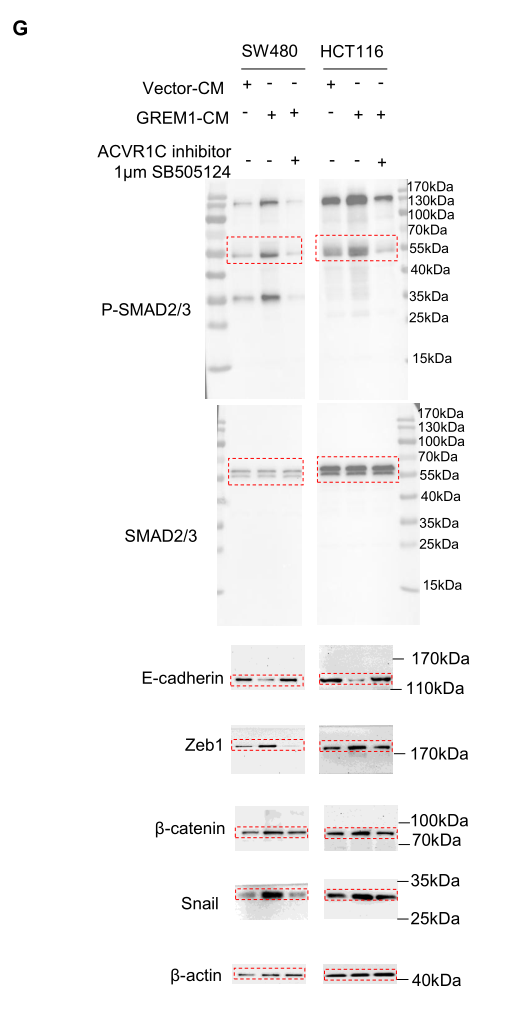


**Figure 6:**


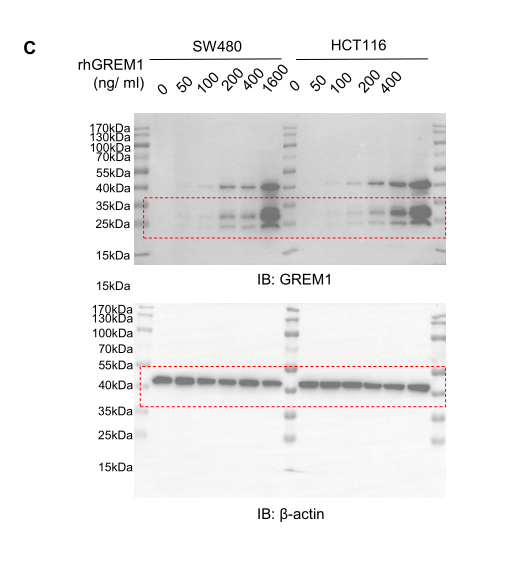


**Figure 6:**


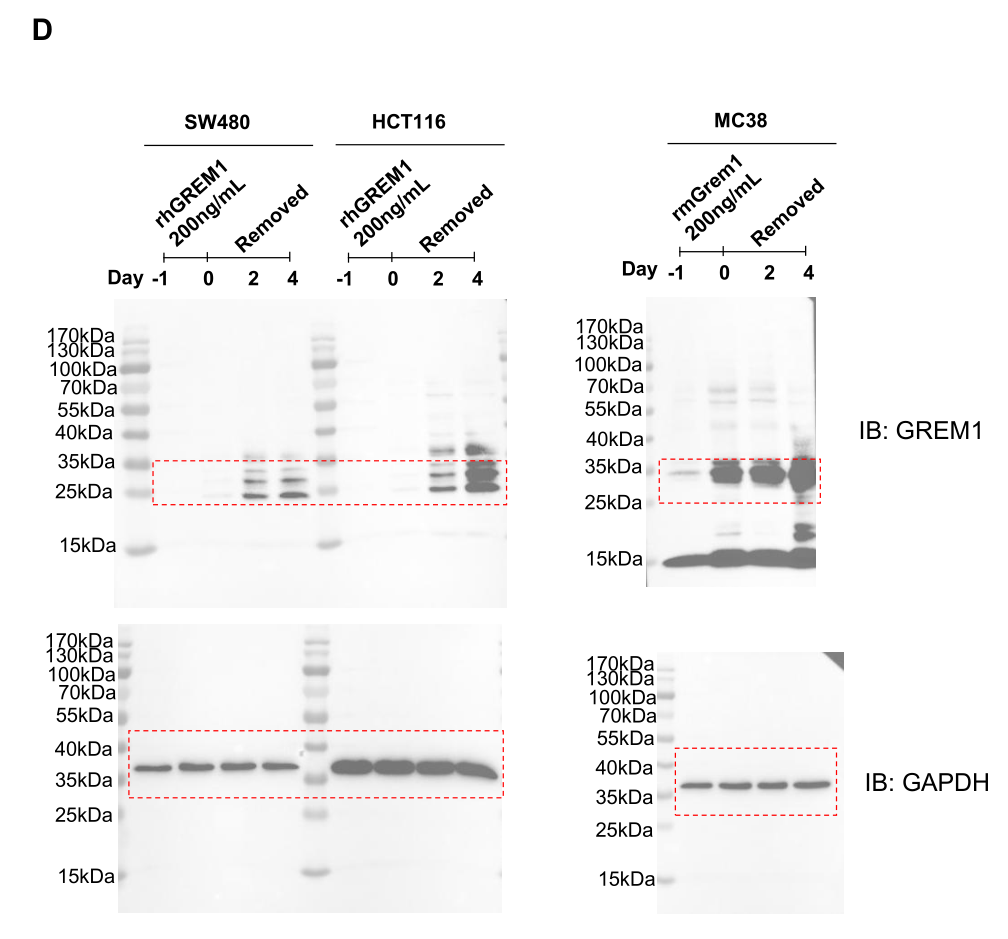


**Supplementary figure 4:**


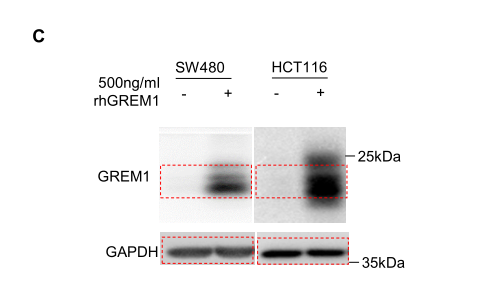


**Supplementary figure 7:**


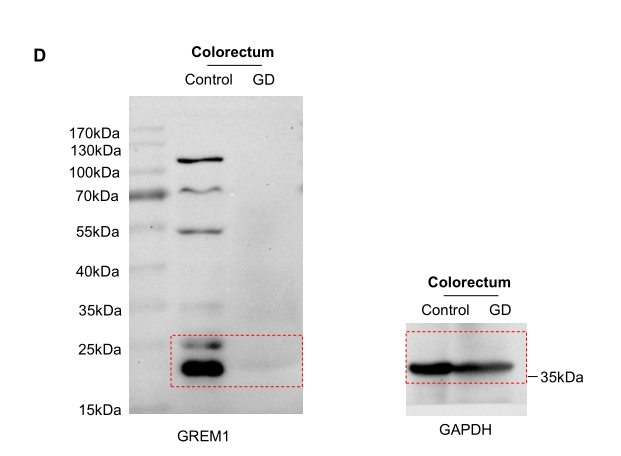

Supplement: Supplementary file 5 — Supplementary Material 5. [file 12943_2025_2554_MOESM5_ESM.docx]
